# Expanding the Family of Monosubstituted 15-Membered Pyridine-Based Macrocyclic Ligands for Mn(II) Complexation in the Context of MRI

**DOI:** 10.1021/acs.inorgchem.5c00452

**Published:** 2025-04-11

**Authors:** Marie Pražáková, Daouda Ndiaye, Éva Tóth, Bohuslav Drahoš

**Affiliations:** † Department of Inorganic Chemistry, Faculty of Science, Palacký University Olomouc, 17. listopadu 12, 771 46 Olomouc, Czech Republic; ‡ Centre de Biophysique Moléculaire, CNRS-UPR 4301, Université d’Orléans, rue Charles Sadron, 45071 Orléans, France

## Abstract

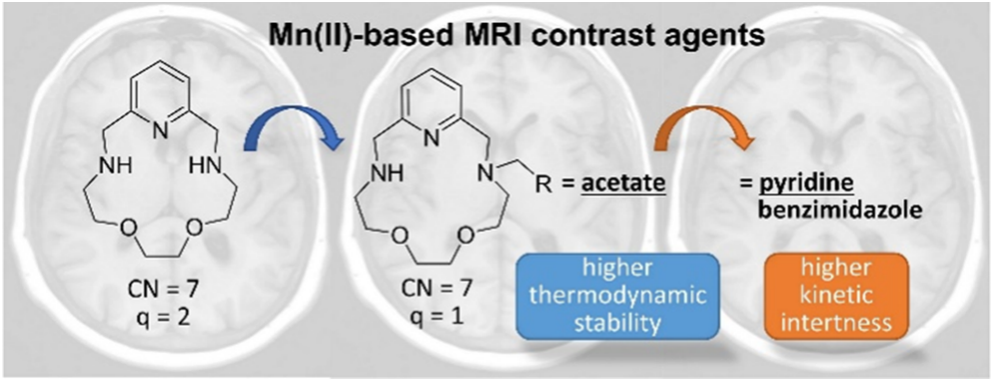

As Mn(II) complexes attract continuous interest as alternatives to Gd-based contrast agents (CAs) in clinical magnetic resonance imaging (MRI), we synthesized two monosubstituted derivatives of the 15-membered pyridine-based macrocycle 15-pyN_3_O_2_ bearing either a 2-pyridylmethyl (**L2**) or a 2-benzimidazolylmethyl pendant arm (**L3**) and characterized their Mn(II) complexes **MnL2** and **MnL3** in the context of MRI contrast agent development. Their X-ray molecular structures confirmed a coordination number of seven and a pentagonal bipyramidal geometry with one coordination site available for inner-sphere water. Protonation constants of **L2** and **L3**, and stability constants with selected divalent metal ions were determined using potentiometry. **MnL2** and **MnL3** complexes are fully formed at pH 7.4; however, they both display low kinetic inertness due to a significant spontaneous dissociation of the nonprotonated complex. The presence of one inner-sphere water molecule in the Mn(II) complexes was confirmed by ^17^O NMR and ^1^H NMRD measurements. The water exchange rate constants are very low (*k*_ex_^298^ = 0.46 × 10^7^ and 0.23 × 10^7^ s^–1^ for **MnL2** and **MnL3**, respectively), but typical for Mn(II) complexes of 15-pyN_3_O_2_ derivatives. The relaxivities are in good agreement with monohydrated small-molecular-weight Mn(II) chelates (*r*_1_ = 2.49 and 2.77 mM^–1^ s^–1^ at 20 MHz, 25 °C, for **MnL2** and **MnL3**, respectively).

## Abstract 2

Since Mn(II) complexes are continuously being explored as alternatives to Gd-based contrast agents in clinical magnetic resonance imaging (MRI), we synthesized two monosubstituted derivatives of the 15-membered pyridine-based macrocycle 15-pyN_3_O_2_ bearing either a 2-pyridylmethyl (**L2**) or a 2-benzimidazolylmethyl pendant arm (**L3**) and characterized their Mn(II) complexes **MnL2** and **MnL3** in the context of MRI contrast agent development.

## Introduction

Magnetic resonance imaging (MRI) is one of the most important noninvasive imaging modalities, which requires the use of a strong magnetic field combined with radio frequency waves to generate three-dimensional (3D) images.^1,2^ To improve image quality or to better visualize tissue perfusion, paramagnetic contrast agents (CAs) are often required.^3^ The efficiency of CAs is commonly described by their relaxivity, *r*_1_, defined as the enhancement of the longitudinal water proton relaxation rate in the presence of a 1 mM concentration of the paramagnetic probe. All CAs used in clinical practice are based on coordination compounds which consist of acyclic or macrocyclic ligands complexing Gd(III). Gd(III) possesses a symmetric electronic ground state with seven unpaired electrons, the highest electron spin for a metal ion (*S* = 7/2), and thus high relaxivity values.^4^ However, nephrogenic systemic fibrosis (NSF), which affects different parts of the body and can be fatal, has been associated with gadolinium injections. As a consequence, today the use of contrast agents is not allowed for kidney-impaired patients.^5,6^ It has been also shown that repeated intravenous administration of MRI CAs may lead to a risk of brain or bone deposition of gadolinium.^7^ Lately, concerns are also raising about the ecological impact of Gd-based MRI agents, which become detectable in natural waters around the world.^8^

Considering these health and environmental risks, the search for more biocompatible nongadolinium CAs has become an active research area. Complexes of paramagnetic transition metal ions have been investigated as alternatives. The most promising is Mn(II), endowed with five unpaired electrons and slow electronic relaxation making it an efficient relaxation agent.^9,10^ The effect of the lower electron spin number, vs Gd(III), on relaxivity might be compensated by the typically shorter Mn–H_water_ bond distance in Mn(II) complexes in comparison to the Gd–H_water_ distance.^11^ Importantly, manganese is an essential element present as a cofactor in several enzymes. Living organisms can thus handle some free Mn(II); nevertheless, in a large excess, it can lead to a neurodegenerative disorder called “manganism” with Parkinson-like symptoms.^12^ This implies that Mn(II) must be strongly chelated for *in vivo* use. Other potential contrast agent alternatives include Fe(III) complexes (*S* = 5/2) which lately attracted increasing attention.^13−15^

**Scheme 1:**
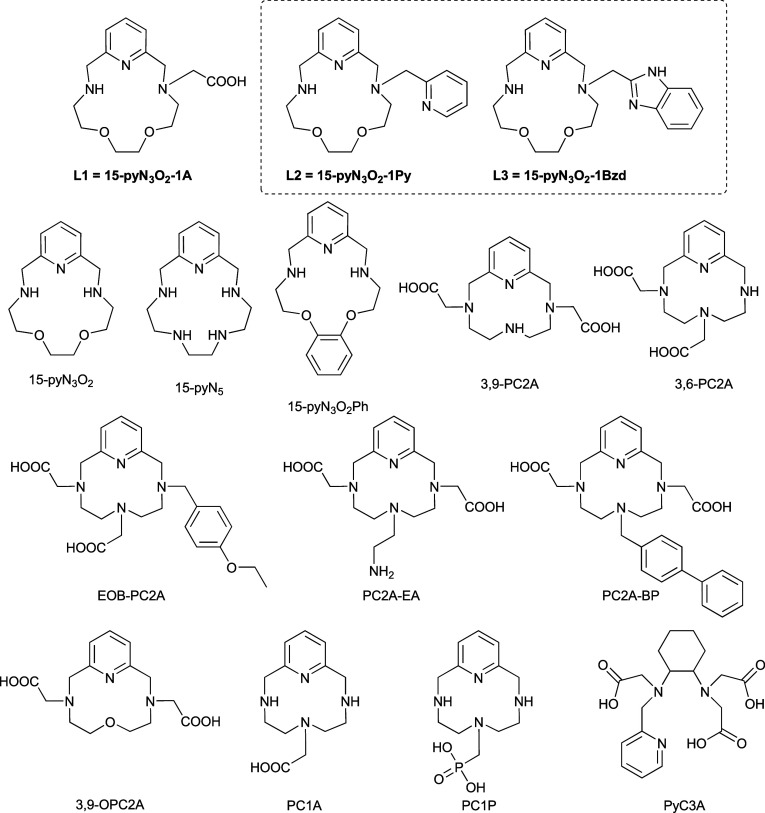
Structural Formulas of Investigated Ligands **L1**–**L3** and Other Relevant Ligands Discussed in the Text

Generally, penta- or hexadentate ligands are used for the complexation of Mn(II) resulting in coordination number (CN) 6 or 7 with one inner-sphere water molecule (*q* = 1).^9^ This metal-coordinated water molecule is required in order to achieve good relaxivity values since its exchange with bulk water transfers the paramagnetic effect to the bulk, resulting in MRI-detectable changes. Maintaining thermodynamically stable and kinetically inert Mn(II) complexation, in addition to inner-sphere water coordination, is often challenging. Although some acyclic chelators are very promising (MnPyC3A, Scheme 1, is currently in clinical trials),^16^ macrocyclic ligands tend to be more suitable in this respect. The benefits of using macrocyclic coordination cavities involve generally higher thermodynamic stability, kinetic inertness, and the opportunity to fine-tune the coordination environment through functionalization, e.g., via the introduction of pendant arms.^17,18^ The presence of a pyridine ring in the macrocyclic scaffold further rigidifies the complex, resulting in increased thermodynamic stability and kinetic inertness. Such pyridine-based ligands of various sizes and with different functional pendant groups appended on the macrocycle *N*-donor atoms are extensively studied for Mn(II) complexation.^4,9,19^

The most common pyridine-based macrocycles are pyclen derivatives.^4,9,17^ In 2011, we synthesized and studied monofunctionalized ligands PC1A and PC1P (Scheme 1) bearing a single acetate or phosphonate pendant arm. They both form six-coordinate Mn(II) complexes containing one inner-sphere water molecule (CN = 6; *q* = 1) which was confirmed with X-ray crystal structures as well. Unfortunately, these complexes are air-sensitive and undergo oxidation to the Mn(III) form.^20^ The bisfunctionalized pyclen-based macrocycles 3,6-PC2A and 3,9-PC2A (Scheme 1) and their derivatives proved to be better suited for Mn(II) chelation in the context of MRI. Their Mn(II) complexes are seven-coordinated, sufficiently stable, kinetically inert, and have reasonable relaxivity values, though the regioisomers differ in many physicochemical aspects.^21^ Other pyclen derivatives, such as PC2A-EA, PC2A-BP and EOB-PC2A and their Mn(II) complexes were studied as well.^22−24^ The suitability of 3,9-PC2A led to the design and the synthesis of a series of bifunctional chelators with different metal binding moieties (acetate, α-methyl acetate, and amide) and reactive (nitro or carboxylate) groups.^25^ These bifunctional chelators were then coupled to anti-HER2 antibody and the Mn(II) complexes were studied as targeted PET/MRI agents.^25^ 3,9-OPC2A (Scheme 1) is a less basic analogue of 3,9-PC2A containing one nonprotonable O atom in the macrocyclic scaffold.^26^ The presence of the O atom does not significantly influence the stability nor relaxivity of the Mn(II) complex; however, the acid-assisted dissociation becomes considerably slower. Regarding other structures, Mn(II) complexes with bispidine derivatives are also extensively studied due to their extraordinary inertness and potential selectivity over Zn(II) related to their highly preorganized and rigid structure.^27^

In the quest of finding an appropriate balance between complex stability, the presence of coordinated water molecule(s), and high relaxivity, 15-membered macrocycles containing a pyridine unit in the scaffold have also been intensively studied. Their Mn(II) complexes are expected to have a pentagonal bipyramidal structure with an overall coordination number of 7 and potential water coordination sites above and below the ligand plane. In 2010, we reported the Mn(15-pyN_5_) and Mn(15-pyN_3_O_2_) complexes (Scheme 1) which indeed contained two inner-sphere water molecules (CN = 7; *q* = 2).^28^ While Mn(15-pyN_5_) combined sufficient stability with high relaxivity thanks to the two inner-sphere water molecules, Mn(15-pyN_3_O_2_) was thermodynamically less stable due to two O-donor atoms in the scaffold, and its kinetic inertness was too low. More recently, an *ortho-*phenylene-derivative ligand, 15-pyN_3_O_2_Ph (Scheme 1) was synthesized.^29^ Its Mn(II) complex was surprisingly flat, with two inner-sphere water molecules (*q* = 2) resulting in good relaxivity; however, its very low thermodynamic and kinetic stabilities prevented further investigations.

Recently, we functionalized one of the macrocyclic nitrogen atoms of 15-pyN_3_O_2_ with an acetate pendant arm (15-pyN_3_O_2_-1A, **L1**; see Scheme 1).^30^ This modification led to increased thermodynamic stability and kinetic inertness for the Mn(II) complex in comparison with the parent macrocycle 15-pyN_3_O_2_, yet the relaxivity values decreased since **MnL1** contains only one inner-sphere water molecule. In an effort to combine improved Mn(II) complex stability and relaxation efficiency within this family of 15-membered pyridine-based macrocycles, herein, we explore other derivatives and report two novel chelators 15-pyN_3_O_2_-1Py (**L2**) and 15-pyN_3_O_2_-1Bzd (**L3**) bearing one pyridine or one benzimidazole pendant arm (Scheme 1), respectively. The aim was to assess the effect of these coordinating functions in the pendant arm on thermodynamic stability, kinetic inertness, as well as the microscopic parameters that govern the relaxivity of the corresponding Mn(II) complexes. The molecular structures of **MnL2** and **MnL3** were determined by single-crystal X-ray diffraction analysis, confirming expected CN = 7 and a pentagonal bipyramidal geometry for both complexes. The molecular structures of the **CuL2** and **CuL3** analogues were also obtained for comparative purposes. Ligand protonation constants and thermodynamic stability constants of Ca(II), Mn(II), Zn(II), and Cu(II) complexes have been measured by potentiometry and they confirm the good coordination capacity of the pyridine and the benzimidazole functions in these complexes. The kinetic inertness of **MnL2** and **MnL3** has been assessed in Zn^2+^ and Cu^2+^ transmetalation experiments using *T*_2_ relaxation time and UV–vis measurements, respectively. A combined ^1^H NMRD and ^17^O NMR study allowed us to assess all of the parameters that determine the relaxivity for the monohydrated (*q* = 1) **MnL2** and **MnL3**. Finally, the redox properties of both Mn(II) complexes have been investigated by cyclic voltammetry in CH_3_CN as well as in aqueous solution, confirming the stability of the Mn(II) oxidation state.

## Experimental Section

### Materials and Methods

**Scheme 2:**
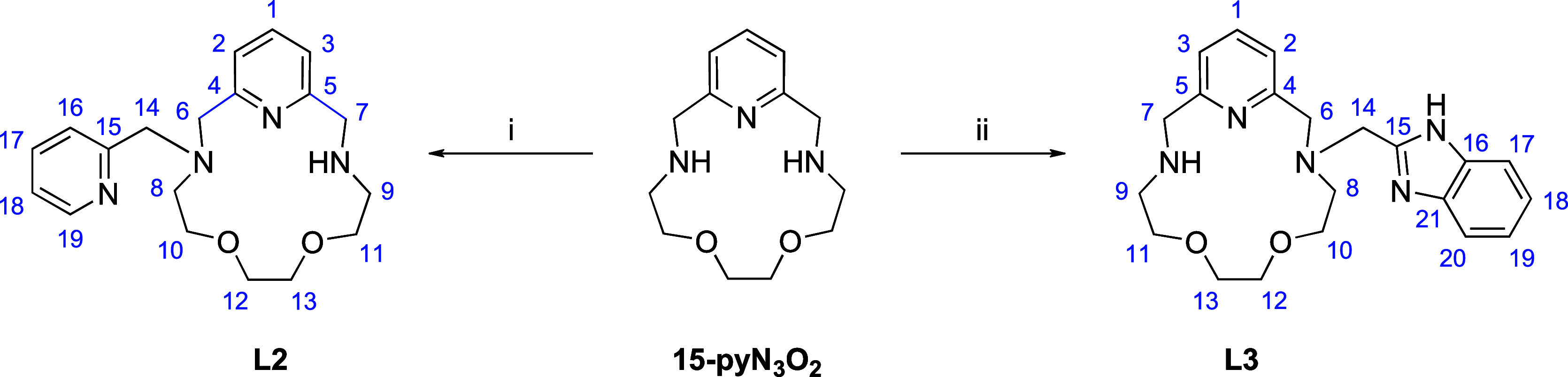
Synthetic Scheme for Ligands **L2** and **L3** and Atom Numbering Applied for Assignment of Signals in NMR Spectra (i) 2-(Chloromethyl)pyridine hydrochloride, NaI, NaHCO_3_, CH_3_CN, RT; (ii) 2-(chloromethyl)benzimidazole, NaI, NaHCO_3_, CH_3_CN, RT.

All solvents (VWR International, Penta) and chemicals were purchased from commercial sources (Acros Organics, Merck) and used as received. 2-(Chloromethyl)pyridine hydrochloride and 2-(chloromethyl)benzimidazole were prepared according to the previously described procedures.^31,32^ The parent ligand 15-pyN_3_O_2_ was synthesized as previously described in the literature.^28,33^ Mass spectra were collected using an LCQ Fleet mass spectrometer (Thermo Scientific, Waltham, MA) equipped with an electrospray ion source and a three-dimensional (3D) ion-trap detector in the positive/negative mode (see SI, Figures S1 and S2). ^1^H and ^13^C NMR spectra were obtained using a 400-MR NMR spectrometer (Varian, Palo, Alto, CA) at 25 °C. The multiplicity of the signals is indicated as follows: s, singlet; d, doublet; t, triplet; m, multiplet; and bs, broad singlet. Deuterated solvent chloroform-*d* (99.9% of D) was purchased from Sigma-Aldrich and used as received. The atom numbering schemes used for the interpretation of NMR data are shown in Scheme 2. The carbon and hydrogen atoms were assigned according to the spectra obtained from the two-dimensional correlation experiments ^1^H–^1^H *gs*-COSY, ^1^H–^13^C *gs*-HMQC, and ^1^H–^13^C *gs*-HMBC (see SI, Figures S3–S10). Cyclic voltammetry was performed on a CHI600C electrochemical analyzer (CH Instruments, Inc., Austin, TX). IR spectra were measured on a Jasco FT/IR-4700 spectrometer (Jasco, Easton, MD) using the ATR technique on a diamond plate in the spectral range 4000–400 cm^–1^ (see SI Figure S11). A conventional electrochemical three-electrode-type cell with a glassy carbon working electrode, a platinum wire auxiliary electrode, and either Ag/Ag^+^ reference electrode (0.01 M AgNO_3_ in 0.1 M tetrabutylammonium perchlorate (TBAP) in CH_3_CN) or Ag/AgCl reference electrode filled with 3 M KCl solution was used during the measurements with the scan rate of 100 mV s^–1^. The measurements were performed in an argon atmosphere either in 0.1 M TBAP solution in CH_3_CN or in 0.15 M aqueous solution of KCl as a supporting electrolyte with a scan rate of 100 mV s^–1^ and at 1.5 mM concentration of the complexes. The internal ferrocene/ferrocenium standard (*E*_1/2_ = 0.459 V vs Ag/Ag^+^ electrode, Δ*E* = 77 mV) was employed for measurements in CH_3_CN.

### Synthesis of **L2**

15-pyN_3_O_2_ (1.2 g, 4.78 mmol) was dissolved in dry CN, and NaI (0.43 g, 2.87 mmol) together with NaHCO_3_ (0.5 g, 5.95 mmol) was added to this mixture. 2-(Chloromethyl)pyridine hydrochloride (0.35 g, 2.14 mmol) was added portionwise, and the reaction mixture was stirred 48 h at room temperature. The reaction mixture was filtered via a glass frit, and the filtrate was evaporated to dryness under reduced pressure. The crude product containing the mixture of disubstituted parent macrocycle, **L2**, and unreacted **15-pyN**_**3**_**O**_**2**_ was purified using column chromatography (SiO_2_, 40–63 μm, pore size 60 Å; EtOH/NH_3_ (aq.) 50:1 → 25:1) and dried *in vacuo* at 50 °C giving an orange oil (240 mg, 32.9%). ^1^H NMR (400 MHz, CDCl_3_): δ 8.55 (dd, *J* = 3.9 and 0,8 Hz, 1H, H19); 7.69–7.63 (m, 1H, H17); 7.58–7.52 (m, 2H, H1/H16); 7.19–7.13 (m, 1H, H18); 7.05 (d, ^2^*J*_HH_ = 7.8 Hz, 2H, H3/H2); 3.90 (d, *J* = 2.4 Hz, 4H, H14/H7); 3.87 (s, 2H, H6); 3.81 (t, *J* = 7.0 Hz, 2H, H10); 3.69–3.65 (m, 2H, H11); 3.65–3.61 (m, 2H, H12); 3.60–3.55 (m, 2H, H13); 2.90–2.84 (m, 4H, H8/H9). ^13^C NMR (400 MHz, CDCl_3_): δ 159.66 (C15); 158.41 (C5); 157.52 (C4); 149.06 (C19); 136.43 (C17); 136.32 (C1); 123.19 (C16); 121.97 (C2/C18); 120.63 (C3); 70.87 (C13); 70.22 (C12); 70.04 (C11); 69.05 (C10); 61.53 (C14); 59.00 (C6); 54.40 (C7); 52.89 (C8); 48.93 (C9). MS *m*/*z* (+): 343.17 ([L2 + H^+^]^+^ calcd 343.21), 365.16 ([L2 + Na^+^]^+^ calcd 365.20) (Figures S1 and S3–S6).

### Synthesis of **L3**

15-pyN_3_O_2_ (2.0 g, 7.96 mmol) was dissolved in dry CN, and NaI (0.6 g, 4.00 mmol) together with NaHCO_3_ (0.78 g, 9.14 mmol) was added to this mixture. 2-(Chloromethyl)benzimidazole (0.7 g, 4.21 mmol) was added portionwise, and the reaction mixture was stirred 48 h at room temperature. After 48 h, the reaction mixture was filtered via a glass frit and the filtrate was evaporated to dryness under reduced pressure. The oily orange residue was dissolved in water containing KOH and filtered via a glass frit. The filtrate was extracted with chloroform (4 × 60 mL), and the combined organic layers were dried over MgSO_4_ and filtered off. Dried organic layers were evaporated to dryness under reduced pressure, and the pure **L3** was obtained in the form of white powder (384 mg, 24%) after recrystallization from CH_3_CN. ^1^H NMR (400 MHz, CDCl_3_): δ 7.62 (bs, 1H, H17/H20); 7.50 (t, ^3^*J*_HH_ = 7.4 Hz, 2H, H1+H20/H17); 7.16 (dd, *J* = 5.9 and 3.1 Hz, 2H, H18/H19); 7.02 (d, ^3^*J*_HH_ = 7.4 Hz, 2H, H); 4.02 (s, 2H, H14); 3.91 (br., 4H, H7/H6); 3.84 (d, *J* = 4.3 Hz, 2H, H11); 3.72 (d, *J* = 4.3 Hz, 2H, H13); 3.64–3.57 (m, 2H, H12); 3.57–3.51 (m, 2H, H10); 2.91 (d, *J* = 3.9 Hz, 4H, H8/H9). ^13^C NMR (400 MHz, CDCl_3_): δ 157.86 (C4); 155.11 (C15); 136.63 (C1); 121.27 (C18/C19); 120.85 (C2/C3); 118.55 (C17 or C20); 111.20 (C17 or C20); 70.23 (C12/C13); 70.10 (C12/C13); 68.92 (C10); 60.99 (C11); 55.93 (C8); 54.13 (C7); 52.86 (C14); 49.21 (C9). MS *m*/*z* (+): 382.16 ([L3 + H^+^]^+^ calcd 382.22), 404.17 ([L3 + Na^+^]^+^ calcd 404.20) (Figures S2 and S7–S10).

### Crystal Structure Determination

**Figure 1:**
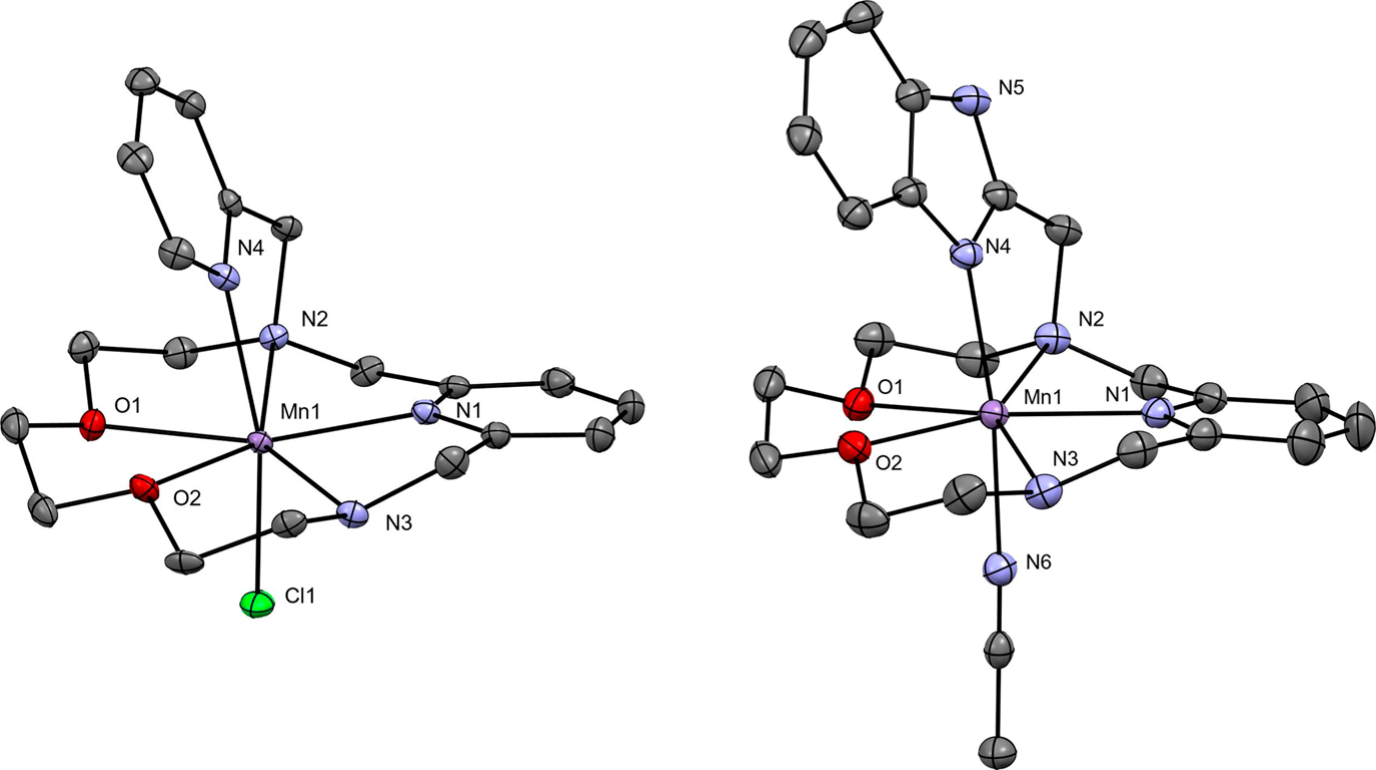
Molecular structures of [Mn**L2**Cl]^+^ (*left*) found in the crystal structure of [Mn**L2**Cl]_2_[MnCl_4_]·DMF and the molecular structure of [Mn**L3**(CN)]^2+^ found in the crystal structure of [Mn**L3**(CN)](ClO_4_)_2_·(CH_3_)_2_CO (*right*). Hydrogen atoms are omitted for clarity. The thermal ellipsoids are drawn at the 50% probability level.

**Figure 2:**
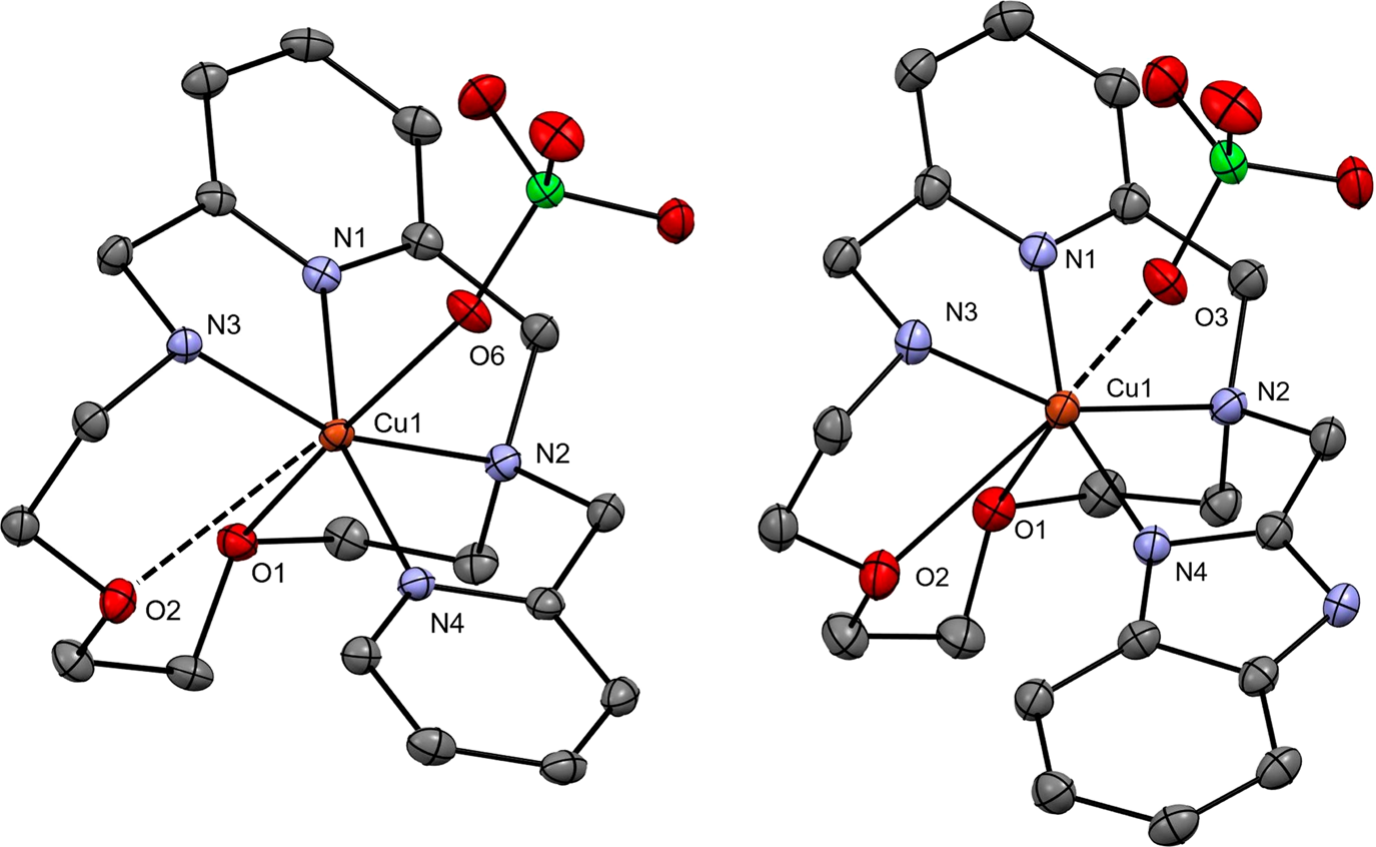
Molecular structures of the [Cu**L2**(ClO_4_)]^+^ complex (*left*) found in the crystal structure of [Cu**L2**(ClO_4_)](ClO_4_)·2CN and molecular structure of the [Cu**L3**(ClO_4_)]^2+^ (*right*) complex found in the crystal structure of [Cu**L3**(ClO_4_)](ClO_4_). Hydrogen atoms are omitted for clarity. Dashed lines represent semicoordination. The thermal ellipsoids are drawn at the 50% probability level.

Single crystals suitable for X-ray diffraction were prepared by mixing a methanol/acetonitrile solution of ligands **L2** or **L3** (*c*_L_ = 36.75 mmol) and manganese(II) perchlorate or manganese(II) chloride (*c*_Mn_ = 35 mM) in a 1:1.05 Mn/ligand ratio. Several drops of But_3_N were added to the ligand solution to adjust the pH to 7 right before the addition of the manganese(II) salt solution. The resulting mixture was then filtered off and evaporated under reduced pressure. The residue containing **MnL2** was dissolved in a CN/iPrOH/OH mixture, several drops of DMF were added to the solution, and the whole mixture was left to evaporate at room temperature leading to colorless crystals. As for **MnL3**, the residue was dissolved in a OH/CN mixture containing a small amount of acetone, and colorless crystals were obtained after slow diffusion of diethyl ether vapors at 5 °C into the solution. Crystals containing Cu(II) were prepared by mixing a corresponding ligand methanol/acetonitrile solution of **L2** and **L3**, (c_L_ = 33 mM) and copper(II) perchlorate (32 mM) in a 1.03:1 ligand/Cu molar ratio. The resulting solution was then stirred at 50 °C and filtered off. The residue was evaporated under reduced pressure and dissolved in a small amount of methanol/acetonitrile solution and dark blue crystals of **CuL2** and **CuL3** were obtained after slow diffusion of diethyl ether vapors at 5 °C into the solution. An identical procedure was also applied for the preparation of single crystals of **CuL2-Boc**. X-ray diffraction data were collected on an XtaLAB Synergy-i (Rigaku) diffractometer equipped with a HiPix3000 Bantam detector and a monochromatized microfocus PhotonJet-i Cu Kα radiation source (λ = 1.54184 Å) at 100 K. The molecular structure of the complex was solved by direct methods and refined by full-matrix least-squares based on F2 (SHELXL 2014/07).^34^ Program Olex2 1.3 was used for the final refinement.^35^ The hydrogen atoms on carbon atoms were fixed into idealized positions (riding model) and assigned temperature factors either *H*_iso_(H) = 1.2*U*_eq_ (pivot atom) for CH and CH_2_ moieties or *H*_iso_(H) = 1.5*U*_eq_ (pivot atom) for CH_3_ groups. The crystal structures of the Mn(II) and Cu(II) complexes of **L2** and **L3**, depicted in Figures 1, 2 and S13–S15, were drawn using Mercury software.^36^

### Thermodynamic Stability Studies

Metal stock solutions were prepared from the highest analytical grade chemicals, and their concentration was determined by complexometric titration with standardized Na_2_H_2_EDTA, using xylenol orange indicator in the presence of urotropine buffer (ZnCl_2_ and MnCl_2_); Eriochrome black T indicator in the presence of ammonium acetate buffer (CaCl_2_) or murexide indicator in ammonium solution (CuCl_2_). The concentration of the ligand stock solutions was determined by pH-potentiometric titrations (the difference between two consecutive inflection points of the ligand titration curve corresponds to two (**L2**) or one (**L3**) ligand equivalents). To determine the protonation constants of the ligand and stability constants of the metal complexes with selected divalent metal ions, pH-potentiometric titrations were carried out with 0.1 M NaOH at 2 mM ligand concentration, in the absence (for ligand protonation constants) or in the presence of one equivalent of metal ion (for stability constants). During the titration, the waiting time was 2 min between successive points (*V*_0_ = 5 mL). The temperature (25.0 ± 0.1 °C) was controlled by a circulating water bath. The ionic strength was set to *I* = 0.15 M NaCl. All titrations were performed under a nitrogen atmosphere to avoid the effect of CO_2_. Titrations were carried out in the pH range from 1.8 (**L3**) or 2.0 (**L2**) (after the addition of 1 M HCl solution to the starting ligand solution in the titration cell) to pH 11 (or until precipitation of the metal hydroxide). All pH-potentiometric titrations were performed using a 785 DMP Titrino titration workstation with a combined electrode (Metrohm). The *p*[H] (*p*[H] = −log[H+], concentration in molarity) was measured in each titration with a combined pH glass electrode (Metrohm) filled with 3 M KCl. The electrode was calibrated in hydrogen ion concentration by titration of HCl with KOH in 0.1 M electrolyte solution.^37^ A plot of potential versus *p*[H] allows determination of the electrode standard potential (*E°*) and the slope factor (*f*).

The protonation constants of the ligand (log *K_i_*^H^) are defined as follows
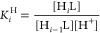
where *i* = 1, 2, ··· and [H*_i_*_–1_L] and [H^+^] are the equilibrium concentrations of the ligand (*i* = 1), its protonated forms (*i* = 2, ···), and the hydrogen ion. The stability (*K*_ML_) and the protonation (*K*_MHiL_) constants for the metal complexes are defined as follows
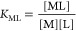

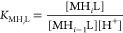
For the calculations of the equilibrium constants, the *HYPERQUAD* program was used.^38^ The number of fitted data pairs was between 190 and 240. The ionic product of water at 25 °C and 0.1 M ionic strength is p*K*_w_ = 13.77.^39^ Fixed values were used for p*K*_w_, ligand acidity constants, and total concentrations of metal, ligand, and acid. All values and errors (one standard deviation) reported are at least the average of two independent experiments.

### Sample Preparation

**MnL2** and **MnL3** complex samples for kinetic and relaxometric studies have been prepared by mixing MnCl_2_ and **L2** or **L3** solutions of known concentrations in a 1:1.05 ratio and by adjusting the pH to 7.4. Mn(II) concentration in each sample was verified by bulk magnetic susceptibility measurements. Both **MnL2** and **MnL3** proved to be water-soluble at the concentrations required for our measurements.

### Kinetic Studies

The transmetalation reaction of **MnL2** and **MnL3** (1.4 mM) was monitored in the presence of 10, 20, 30, and 40 mol equiv of Zn(II) ions, by measuring water proton *T*_2_ relaxation times (60 MHz) as a function of time at 25 °C and in 0.15 M NaCl, in the pH range 4.9–6.0 (4.9, 5.0, 5.2, 5.5, and 6.0). To control pH, 50 mM *N*-methyl-piperazine (pH 4.9–5.5) or 50 mM MES (2-(4-morpholino)ethane-sulfonic acid) buffer (pH 5.7–6.0) was used. Transmetalation of **MnL2** (0.3 mM) was also investigated in the presence of 10, 20, 30, and 40 mol equiv of Cu(II) ions, by monitoring the UV–vis absorbance at 274.0 nm as a function of time at 25 °C and in 0.15 M NaCl, in the pH range 4.7–5.0 (in 75 mM 1,4-dimethyl piperazine (DMP) buffer). No oxidation of the samples was observed. The program OriginPro 9 was used for the fitting of experimental data.

### Relaxation Properties

At 60 MHz, ^1^H longitudinal (*T*_1_) and transverse (*T*_2_) relaxation times were measured with a Bruker Minispec MQ-60 NMR analyzer. The temperature was set to 25.0 °C and controlled with a circulating water bath. *T*_1_ and *T*_2_ values were determined, respectively, by using the inversion recovery method (180−τ–90°) and the Carl–Purcell–Meiboom–Gill (CPMG) spin–echo pulse sequence. The pH-dependent relaxivities (pH 2.1–9.9) were acquired at 1.5 mM **MnL2** and **MnL3** concentration, and the pH was adjusted by 0.045 M MES, HEPES (4-(2-hydroxyethyl)piperazine-1ethanesulfonic acid), DMP, or NMP (*N*-methyl piperazine) buffers.

^1^H NMRD profiles on aqueous solutions of **MnL2** (*c*_Mn2+_ = 0.98 mM; pH = 7.22) and **MnL3** (*c*_Mn2+_ = 0.96 mM; pH = 7.20) were recorded at 25 and 37 °C on a SMARTracer Fast Field Cycling NMR relaxometer (Stelar; 0.00024–0.24 T, 0.01–10 MHz ^1^H Larmor frequency) and a WP80 NMR electromagnet adapted to variable-field measurements (Bruker; 0.47–1.88 T, 20–80 MHz ^1^H Larmor frequency). The temperature was maintained by a gas flow and monitored by a VTC91 temperature control unit based on previous calibration with a Pt resistance temperature probe.

### ^17^O NMR Measurements

Variable-temperature ^17^O NMR measurements of aqueous solutions of **MnL2** (*c*_MnL_ = 3.70 mmol·kg^–1^, pH = 7.03) and **MnL3** (*c*_MnL_ = 3.72 mmol·kg^–1^, pH = 7.2) were performed on a Bruker Avance III 400 MHz spectrometer (9.4 T, 54.2 MHz) in the temperature range 5–75 °C. The temperature was calculated after calibration with ethylene glycol and OH. Acidified water (HClO_4_, pH = 3.3) was used as a diamagnetic reference. The ^17^O transverse relaxation times (*T*_2_) were obtained by using the CPMG spin–echo technique. To eliminate susceptibility contributions to the chemical shift, the sample was in a glass sphere placed in a 10 mm NMR tube. To improve the sensitivity, ^17^O-enriched water (11.10% H_2_^17^O, Cortecnet) was added to the solution to yield approximately 1% ^17^O enrichment.

The ^17^O NMR and NMRD data have been analyzed according to the Solomon–Bloembergen–Morgan theory of paramagnetic relaxation.^3^ The least-squares fit was performed using Visualizeur/Optimiseur running on a MATLAB 8.3.0 (R2014a) platform.

## Results and Discussion

### Synthesis

15-pyN_3_O_2_ was prepared according to literature procedures.^28,33^ The first attempts of **L2** and **L3** synthesis were based on the previously described preparation of **L1**,^30^ where the molar ratio of the macrocycle to the alkylating agent was 6:1. However, this procedure did not strictly lead to the single monosubstituted product and unreacted 15-pyN_3_O_2_, but a significant amount of a disubstituted side product was always present. Consequently, syntheses with an even larger molar excess of the parent macrocycle were tested, without significant improvement. Finally, the molar excess was decreased to ca. 2:1 using NaI and NaHCO_3_ as base (Scheme 2). The synthesis was performed at room temperature because higher temperature or reflux led to Schiff base formation (see Scheme S1 in SI) which was confirmed by MS and IR measurements (Figure S11).

**L2** was purified via column chromatography using SiO_2_ with smaller particle size (40–63 μm), but a major part of the product was still in the form of a mixture with the parent macrocycle, since the *R*_f_ values of **L2** and 15-pyN_3_O_2_ were very close (*R*_f_ = 0.35 and *R*_f_ = 0.29, respectively; EtOH/NH_3_ (aq.) 25:1). The column chromatography was performed also with different mobile phases (e.g., EtOH/CHCl_3_/NH_3_ (aq.) 40:20:2 or 40:10:1), but the two compounds were still difficult to separate, which resulted in a reduced yield and a significant amount of 15-pyN_3_O_2_/**L2** mixture. To separate the product from this mixture after chromatography, different strategies were considered. One of them involved two additional steps, which in the end led to a higher yield (+30%). The first step was the reaction of 15-pyN_3_O_2_/**L2** mixture with Boc_2_O providing Boc-protected **L2** (**L2**-Boc) and 15-pyN_3_O_2_ containing two protecting Boc groups (Boc_2_-15-pyN_3_O_2_, Scheme S2 in SI). The retention factors of these Boc-protected products were much different (*R*_f_ = 0.38 and *R*_f_ = 0.95, for **L2**-Boc and Boc_2_-15-pyN_3_O_2_, respectively; CH_3_Cl/CH_3_OH 90:10 → 60:40) in comparison with the 15-pyN_3_O_2_/**L2** mixture, and therefore, it was possible to separate both products by subsequent column chromatography. The **L2**-Boc side product was characterized using ^1^H NMR (Figure S12) at higher temperatures since room-temperature measurements led to increased number of signals due to the presence of stereoisomers. The second step involved the deprotection of **L2**-Boc using TFA/CH_2_Cl_2_ (Scheme S2 in SI). The unreacted 15-pyN_3_O_2_ was recovered by the similar deprotection reaction of Boc_2_-15-pyN_3_O_2_ (see Scheme S2).

Considering **L3** purification, the crude product was washed with basic water since the disubstituted macrocycle side product was insoluble in water. The filtrate was then extracted with CHCl_3_, and the dried organic fractions were evaporated. The residue containing the mixture of **L3** and 15-pyN_3_O_2_ was dissolved in hot CH_3_CN and the solution was slowly cooled down giving **L3** as a white powder, whereas the parent macrocycle remained dissolved because of its better solubility in CH_3_CN.

### Crystal Structures Analysis

For single-crystal preparation, a solution of Mn(II) or Cu(II) salt was mixed with a ligand solution in OH in a 1:1 molar ratio. The solution was then evaporated under reduced pressure, and the residue was dissolved in OH/CN (1/1). Crystallization was induced by slow diffusion of acetone/diethyl ether vapors into these solutions at 5 °C. The crystals of [Mn**L3**(CN)](ClO_4_)_2_·(CH_3_)_2_CO (**2**), [Cu**L2**(ClO_4_)](ClO_4_)·2CH_3_CN (**3**) and [Cu**L3**](ClO_4_)_**2**_ (**4**) were obtained using this procedure. To prepare single crystals of [Mn**L2**Cl]_2_[MnCl_4_]·DMF (**1**), many attempts were made by using various solvents and adding DMF; however, most of them yielded an oil or unsuitable crystals. Crystals suitable for X-ray measurements were obtained only after evaporation at room temperature from a OH/i-PrOH/CN mixture containing several drops of DMF.

Crystal data and structure refinements are listed in Table 1, and the molecular structures of complex cations found in complexes **1**, **2** and **3**, **4** are depicted in Figures 1 and 2, respectively. Selected interatomic distances and angles for all four studied complexes are given in Table 2.

**Table 1:** Crystal Data and Structure Refinements for Mn(II) and Cu(II) Complexes of **L2** and **L3**, Complexes **1–4**

compound	[Mn**L2**Cl]_2_[MnCl_4_]·DMF (1)	[Mn**L3**(CH_3_CN)](ClO_4_)_2_·(CH_3_)_2_CO (2)	[Cu**L2**(ClO_4_)](ClO_4_)·2CH_3_CN (3)	[Cu**L3**](ClO_4_)_**2**_ (4)
formula	C_41_H_59_Cl_6_Mn_3_N_9_O_5_	C_26_H_36_Cl_2_MnN_6_O_11_	C_23_H_32_Cl_2_CuN_6_O_10_	C_21_H_27_Cl_2_CuN_5_O_10_
*M*_r_	1135.49	734.45	686.98	643.91
temperature (K)	99.97(13)	100.00(10)	100.02(11)	100.15
crystal system	orthorhombic	monoclinic	monoclinic	orthorhombic
space group	*Pna*2_1_	*P*2_1_/*n*	*P*2_1_*/n*	*Pccn*
*a* (Å)	17.73810(10)	14.48776(15)	12.51519(9)	23.07724(19)
*b* (Å)	16.67560(10)	10.86824(11)	9.04190(7)	17.34191(14)
*c* (Å)	17.02880(10)	20.1761(2)	25.51611(19)	14.19998(12)
α (°)	90	90	90	90
β (°)	90	91.0696(9)	96.4361(7)	90
γ (°)	90	90	90	90
*V*, Å^3^	5037.01(5)	3176.31(6)	2869.23(4)	5682.88(8)
*Z*	4	4	4	8
*D*_calc_ (g cm^–3^)	1.497	1.536	1.590	1.505
μ (mm^–1^)	9.374	5.519	3.362	3.345
*F*(000)	2340	1524	1420	2648
*R*(int)^a^	0.0296	0.0256	0.0413	0.0247
data/restraints/parameters	8627/1/580	5766/0/418	5212/0/381	5190/0/352
completeness to θ (%)	99.8	98.9	99.3	99.5
goodness-of-fit on *F*^2^	1.030	1.075	1.051	1.055
*R*_1_, *wR*_2_ (*I* > 2σ(*I*))^b^	0.0241/0.0619	0.0334/0.0867	0.0388/0.1068	0.0273/0.0760
*R*_1_, *wR*_2_ (all data)^b^	0.0243/0.0619	0.0370/0.0885	0.0410/0.1091	0.0296/0.0776
largest diff. peak and hole (A^–3^)	0.18 and −0.47	0.54 and −0.41	0.81 and −0.69	0.28 and −0.44
CCDC	2407086	2407087	2407088	2407089

a *R*_int_ = Σ|*F*_0_^2^ – *F*_o,mean_^2^|/Σ*F*_0_^2^.

b *R*_1_ = Σ(||*F*_0_| – |*F*_c_||)/Σ|*F*_0_|; *w*R**_2_ = [Σ*w*(*F*_0_^2^ – *F*_c_^2^)^2^/Σ*w*(*F*_0_^2^)^2^]^1/2^.

**Table 2:** Selected Interatomic Distances [Å] and Aangles [°] in the Mn(II) and Cu(II) Complexes **1–4**

compound	**1**	**2**		**3**	**4**
Distances					
Mn1–N1	2.227(2)	2.226(2)	Cu1–N1	1.933(2)	1.946(1)
Mn1–N2	2.323(2)	2.384(2)	Cu1–N2	2.080(2)	2.100(1)
Mn1–N3	2.301(3)	2.282(2)	Cu1–N3	2.057(2)	2.066(1)
Mn1–N4	2.325(2)	2.219(2)	Cu1–N4	2.004(2)	1.999(1)
Mn1–Cl1/N6	2.4469(8)	2.291(2)	Cu1–O1	2.523(2)	2.520(1)
Mn1–O1	2.354(2)	2.313(1)	Cu1–O2	2.800(2)	2.649(1)
Mn1–O2	2.270(2)	2.280(1)	Cu1–O6/O3	2.662(2)	2.842(1)
Mn2–Cl2	2.4659(8)				
Mn2–O3	2.327(2)				
Mn2–O4	2.264(2)				
Mn2–N5	2.218(2)				
Mn2–N6	2.356(2)				
Mn2–N7	2.298(2)				
Mn2–N8	2.316(2)				
Angles					
N1–Mn1–N2	71.66(8)	71.30(6)	N1–Cu1–N2	82.48(7)	81.36(6)
N1–Mn1–N3	72.00(8)	71.92(6)	N1–Cu1–N3	80.35(7)	80.58(6)
N1–Mn1–N4	95.13(8)	98.76(6)	N1–Cu1–N4	160.70(8)	158.34(6)
N1–Mn1–N6		88.95(6)	N2–Cu1–N3	161.30(7)	158.35(6)
N2–Mn1–N3	143.30(8)	143.16(6)	N2–Cu1–N4	82.73(7)	81.97(6)
N2–Mn1–N4	74.64(9)	75.83(6)	N3–Cu1–N4	112.18(7)	112.09(6)
N2–Mn1–N6		92.05(6)	O1–Cu1–O2		62.68(4)
N3–Mn1–N4	103.74(9)	111.98(6)	O1–Cu1–O6	171.93(5)	
N3–Mn1–N6		85.28(6)	N1–Cu1–O1	83.92(6)	85.80(5)
N4–Mn1–N6		162.53(6)	N2–Cu1–O1	78.65(6)	78.42(5)
O1–Mn1–O2	70.25(7)	71.43(5)	N3–Cu1–O1	106.70(6)	111.95(5)
N1–Mn1–O1	145.21(8)	143.38(6)	N4–Cu1–O1	105.19(6)	104.25(5)
N2–Mn1–O1	74.01(8)	74.16(5)	N1–Cu1–O2		120.45(5)
N3–Mn1–O1	142.64(8)	140.69(6)	N2–Cu1–O2		131.66(5)
N4–Mn1–O1	80.98(8)	84.12(5)	N3–Cu1–O2		68.67(5)
N6–Mn1–O1		80.42(5)	N4–Cu1–O2		81.10(5)
N1–Mn1–O2	144.04(9)	144.73(6)	N1–Cu1–O6	91.90(6)	
N2–Mn1–O2	140.35(8)	143.44(5)	N2–Cu1–O6	93.99(6)	
N3–Mn1–O2	73.53(8)	73.33(6)	N3–Cu1–O6	79.27(6)	
N4–Mn1–O2	83.29(8)	88.64(5)	N4–Cu1–O6	76.84(6)	
N6–Mn1–O2		94.09(6)			
N5–Mn2–N6	71.86(8)				
N5–Mn2–N7	72.30(9)				
N5–Mn2–N8	92.13(9)				
N6–Mn2–N7	143.48(8)				
N7–Mn2–N8	100.30(9)				
N5–Mn2–O3	145.05(8)				
N5–Mn2–O4	143.89(9)				
N6–Mn2–O3	73.63(8)				
N6–Mn2–O4	139.63(8)				
N7–Mn2–O3	142.61(8)				
N7–Mn2–O4	73.10(8)				
N8–Mn2–O3	83.74(8)				
N8–Mn2–O4	84.13(8)				

In the crystal structure of [Mn**L2**Cl]_2_[MnCl_4_]·DMF (**1**), two [Mn**L2**Cl]^+^ units were found in the asymmetric unit, and the positive charge is compensated by a [MnCl_4_]^2–^ counteranion (Figure S13). For each unit, the three nitrogen and two oxygen donor atoms of the macrocycle are coordinated in the equatorial plane, while the nitrogen atom of the pyridine pendant arm and a chloride occupy apical positions, leading to a coordination number of seven for the Mn(II) central atom with pentagonal bipyramidal geometry of the coordination sphere. In aqueous solution, the coordinated chloride is replaced with the oxygen of a water molecule, as it was confirmed by relaxometry and ^17^O NMR (*vide infra*). Among all Mn–N distances, the Mn–N1(pyridine) distance (2.227 Å) is the shortest, and the Mn–N4 distance is the longest (2.325 Å), where N4 is the nitrogen of the pyridine pendant arm (Table 2). The Mn–O distances to the oxygen atoms of the macrocyclic cavity are 2.354 and 2.270 Å (Table 2), thus in the same range as the Mn–N distances. Moreover, in the asymmetric unit of **1**, there is one cocrystallized solvent DMF molecule.

In the crystal structure of [Mn**L3**(CH_3_CN)](ClO_4_)_2_·(CH_3_)_2_CO (**2**), the charge of the [Mn**L3**(CH_3_CN)]^2+^ unit is compensated by two ClO_4_^–^ counteranions. As for the above-described analogue, the coordination sphere has also pentagonal bipyramidal geometry, and one nitrogen donor atom from the benzimidazole pendant arm and one nitrogen atom of an acetonitrile are coordinated in apical positions, leading to the coordination number of seven. Among all Mn–N distances, the longest is Mn–N6 (2.291 Å), where N6 is the nitrogen atom in acetonitrile, and the shortest is again the Mn–N1(pyridine) distance (2.226 Å, Table 2). The acetonitrile coordinated to Mn(II) is replaced in aqueous solution with a water molecule as for the **L2** analogue, again confirmed with relaxometry and ^17^O NMR (*vide infra*). The Mn–O distances to the macrocyclic oxygen atoms are 2.313 and 2.280 Å (Table 2), again comparable to Mn–N distances. In the asymmetric unit of **2**, there is one additional cocrystallized acetone molecule (SI Figure S14), which forms via its oxygen atom a hydrogen bond with the NH group of the benzimidazole pendant arm (N5–H5···O11, 2.802(2) Å, 169.9°).

Crystal structures of Cu(II) complexes with **L2** and **L3** were obtained as well for comparison (Figure 2). In the crystal structure of [Cu**L2**(ClO_4_)](ClO_4_)·2CN (**3**), the coordination sphere of Cu(II) is strongly distorted and consists of four nitrogen donor atoms (macrocycle and pyridine pendant arm, rather short Cu–N distances in the range ∼1.93 to 2.08 Å) and two oxygen atoms, one macrocyclic oxygen atom (O1) and one oxygen atom from the coordinated perchlorate anion with elongated Cu–O distances of 2.523 and 2.662 Å, respectively (Table 2). The coordination sphere is completed with the second macrocyclic oxygen atom (O2) (2.800 Å). The positive charge is compensated by an additional noncoordinated (ClO_4_)^−^ counteranion, and there are also two cocrystallized CH_3_CN molecules originating from the crystal preparation procedure.

The second Cu(II) containing crystal structure [Cu**L3**](ClO_4_)_2_ (**4**) is similar to that of **3**. The coordination sphere of the central Cu(II) atom consists of three nitrogen atoms of the macrocycle and one nitrogen atom of the benzimidazole pendant arm (again, the Cu–N bond distances are rather short in the range ∼1.95 to 2.10 Å, Table 2). The coordination sphere of Cu(II) is completed with two oxygen donor atoms O1 and O2 of the macrocyclic scaffold with elongated bond distances of 2.520 and 2.649 Å, respectively, and with one oxygen atom from the perchlorate anion with a long Cu–O distance, 2.842 Å (Table 2). The positive charge of the complex cation is compensated by one noncoordinated perchlorate anion without any cocrystallized solvent molecules.

Finally, in order to unambiguously confirm the molecular structure of the intermediate **L2**-Boc during the synthesis of **L2**, single crystals of its Cu(II) complex [Cu(**L2**–Boc)(ClO_4_)](ClO_4_).CH_3_CN were also prepared by the same procedure as described above for **CuL2**, and the molecular structure was determined (Figure S15 together with crystal data and structure refinements in Table S1 and selected interatomic distances and angles in Table S2). The central Cu(II) ion is coordinated by two pyridine N atoms, one macrocyclic N atom bearing the pyridine pendant arm, one macrocyclic O atom (the Cu–O1 distance is 2.734 Å), and one O atom of the carbamate moiety. The coordination sphere is completed by a perchlorate anion (Cu–O5 distance is 2.466 Å), and thus, it has strongly axially elongated octahedral geometry in accordance with a strong Jahn–Teller effect. The distance between Cu(II) and the second macrocyclic O atom exceeds 4 Å and thus excludes any bonding interaction.

### Equilibrium Studies in Aqueous Solution

The stepwise protonation constants of **L2** and **L3** and the stability constants formed with Mn(II), Zn(II), Cu(II), and Ca(II) ions have been determined using potentiometric titrations. Complex formation was fast for all systems and allowed for direct titrations. Titration curves are given in SI (Figures S16 and S17) and the calculated constants compared to those of selected macrocyclic ligands are shown in Tables 3 and 4.

**Table 3:** Stepwise Protonation Constants of **L2** and **L3** and Other Selected Ligands Obtained from Potentiometry (*I* = 0.15 M NaCl; 25 °C)

	log *K*_1_^h^	log *K*_2_^h^	log *K*_3_^h^	log *K*_4_^h^
L1^a^	8.86	8.03	1.9	
L2 (this work)	8.58(1)	7.14(1)	2.1(1)	
L3 (this work)	8.50(1)	5.85(1)	1.98(1)	
15-pyN_3_O_2_^b^	8.82	7.80		
15-pyN_5_^b^	9.40^b^ (9.43^c^; 9.11^d^)	8.54^b^ (8.80^c^; 8.82^d^)	5.28^b^ (5.28^c^; 5.27^d^)	
15-pyN_3_O_2_Ph^e^	8.53	7.63		
3,9-PC2A^f^	12.25^f^/12.50^g^	5.97^f^/5.75^g^	3.47^f^/3.28^g^	1.99^f^/2.38^g^
3,6-PC2A^f^	10.72^f^/10.31^h^	8.37^f^/8.59^h^	3.81^f^/3.92^h^	1.26^f^/1.56^h^
PC2A-EA^i^	11.34	8.93	6.91	1.97
PC2A-BP^j^	10.45	6.93	2.36	1.64
3,9-OPC2A^k^	7.73	7.66	2.13	
PC1A^l^	10.47	8.71	2.79	
PC1P^l^	11.84	9.64	6.23	0.99

a Ref (30).

b Ref (28) (0.1 M NMe_4_Cl).

c Ref (40) (0.1 NaClO_4_).

d Ref (41) (0.1 NaClO_4_).

e Ref (29).

f Ref (21).

g Ref (42) (0.1 M KCl).

h Ref (21) (1.0 M NaCl).

i Ref (22).

j Ref (23).

k Ref (26).

l Ref (20) (0.1 M NMe_4_Cl).

**Table 4:**
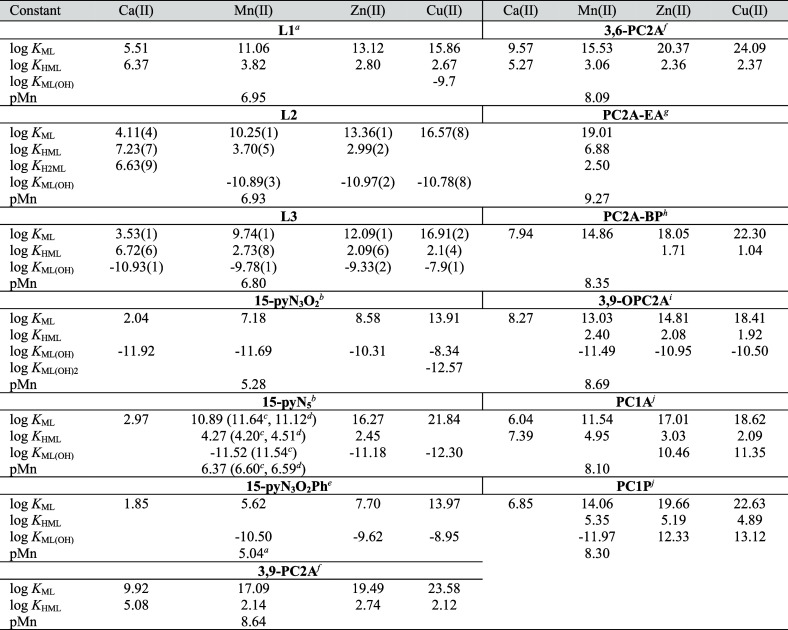
Stability Constants of Ca(II), Mn(II), Zn(II), and Cu(II) Complexes of **L2** and **L3** and Other Selected Ligands (*I* = 0.15 M NaCl; 25 °C) and pMn Values^k^

a Ref (30).

b Ref (28) (0.1 M NMe_4_Cl).

c Ref (40) (0.1 M NaClO_4_).

d Ref (41) (0.1 M NaClO_4_).

e Ref (29).

f Ref (21).

g Ref^22^.

h Ref (23).

i Ref (26).

j Ref (20) (0.1 M NMe_4_Cl).

k pMn = −log[Mn(II)]_free_ at pH 7.4, *c*_Mn_ = *c*_lig_= 10^–5^ M.

The first two protonation constants of **L2** and **L3**, log *K*_1_^H^ and log *K*_2_^H^, correspond to the macrocyclic amines. The values log *K*_1_^H^ ∼ 8.5 of **L2** and **L3** are similar and slightly lower than that observed for the parent 15-pyN_3_O_2_ macrocycle or for the previously described acetate-substituted ligand L1. The second protonation constant, log *K*_2_^H^, decreases in the order of **L1** > **L2 > L3** by about 1 order of magnitude in each case, while the parent macrocycle has a log *K*_2_^H^ value similar to that of **L2**. This trend might be related to the strength of the hydrogen bond established between the protonated macrocyclic N atoms and the pendant arm and/or increasing electron-withdrawing effect of the functional group in order carboxylate < pyridine < benzimidazole^43,44^ (this effect is also apparent from the lower chemical shift of the CH_2_ group in pendant arm, H14 in Scheme 2, for **L2** (3.90 ppm) in comparison with **L3** (4.02 ppm)) providing decrease in basicity of N atom bearing the pendant arm.

The third protonation constant, log *K*_3_^H^, corresponds to the nitrogen protonation of the pyridine or benzimidazole pending arm. Protonation of the macrocyclic pyridine unit is difficult to observe above pH 1.9. When compared to the 15-membered macrocycle with only N donors, 15-pyN_5_, the protonation constants are lower for all oxygen-containing analogues. Related to the *–I* effect of the oxygen atom(s) in the macrocyclic scaffold which leads to lower basicity of the neighboring nitrogen atoms, the same trend is observable in the family of 12-membered macrocycles: the first protonation constant is about 4.5 orders of magnitude lower for 3,9-OPC2A vs pyclen derivatives such as 3,9-PC2A.

According to the stability constants (Table 4), **L2** and **L3** form the most stable complexes with Cu(II), then log *K*_ML_ values decrease in the order Cu(II) > Zn(II) > Mn(II) > Ca(II) following the Irving-Williams series. **ML3** complexes are more prone to hydrolysis, which is observable above pH 9 for Mn(II), Zn(II), and Cu(II). Hydrolysis is present for almost all metal complexes (Table 4, Figures S16 and S17). As expected and previously reported for **L1**, the introduction of an additional coordinating pendant arm on the macrocycle leads to higher stability, even if for Ca(II) or Mn(II) the stabilizing effect of the benzimidazole moiety in **L3** vs 15-pyN_3_O_2_ is less significant. Recently, an empirical approach was proposed to estimate stability constants of MnL complexes based on structural descriptors.^45^ This model provides an estimation of log *K*_MnL_ = 8.5 for **MnL2**, which is lower than the experimental value. On the other hand, no descriptors exist for benzimidazole coordination.

To obtain further information about the impact of a given pendant arm, it is interesting to calculate pMn values (pMn = −log[Mn(II)]_free_ at pH 7.4, *c*_Mn_ = *c*_lig_= 10^–5^ M) which provide a more direct comparison of MnL stabilities, independently of differences in protonation constants (Table 4). The pMn values, thus conditional stability, decrease in the order of **MnL1** > **MnL2** > **MnL3**; however, they remain ∼1.5 magnitude higher for all monosubstituted 15-pyN_3_O_2_ derivatives (**L1**–**L3**) than for the parent 15-pyN_3_O_2_ and comparable to that of the Mn(15-pyN_5_) analogue. Nevertheless, the too large, 15-membered macrocyclic cavity combined with a lower number of pendant arms provide ca. 2 orders of magnitude lower stability for Mn(II) complexation as compared to the 12-membered pyclen derivatives (Table 4).

**Figure 3:**
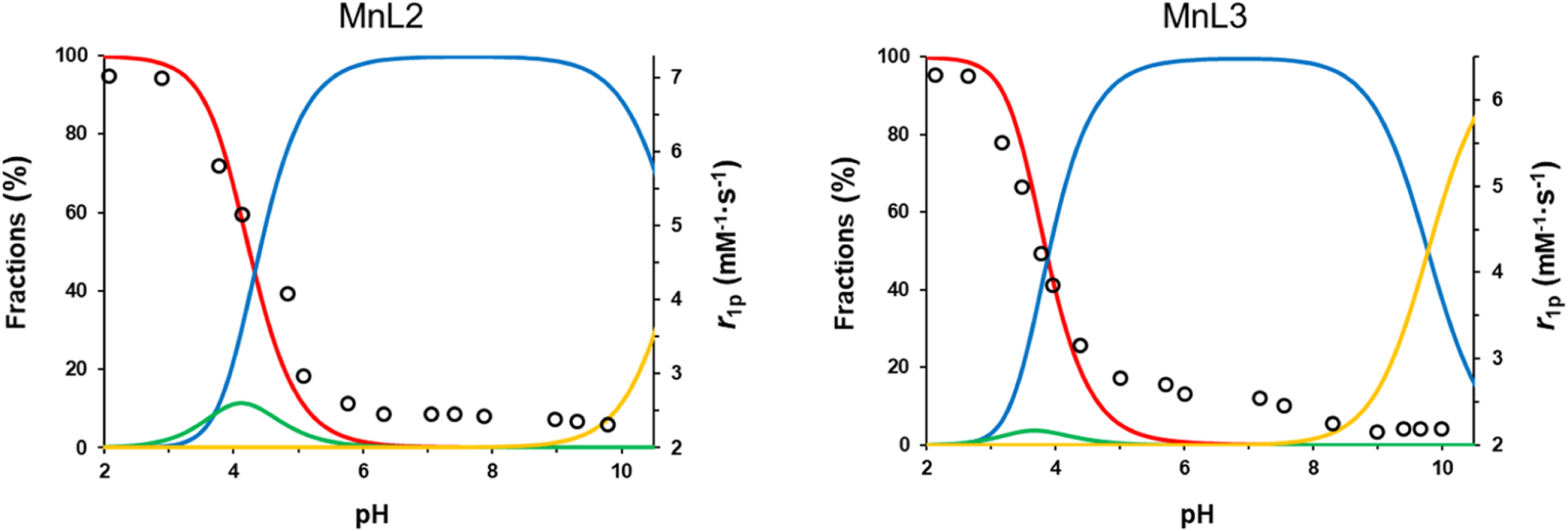
Species distribution diagram of the Mn(II)-**L2**-H^+^ system ([Mn(II)] = [**L2**] = 1.5 mM; solid lines; red = [Mn(II)_free_], green = [MnHL2], blue = [**MnL2**] and yellow = [MnL2H^–1^] (*left*)); and species distribution diagram of the Mn(II)-**L3**-H^+^ system ([Mn(II)] = [**L3**] = 1.5 mM; solid lines; red = [Mn(II)_free_], green = [MnHL3], blue = [**MnL3**], yellow = [MnL3H^–1^] (*right*)); *r*_1p_ obtained as a function of pH at 1.41 T, 60 MHz (*T* = 25 °C and *I* = 0.15 M NaCl; black dots).

The stability constants obtained by pH-potentiometry allowed the calculation of the species distribution curves for **MnL2** and **MnL3**, and these could be corroborated with pH-dependent relaxivity data (Figure 3). In both cases, full complex formation is achieved above pH 6. Below pH 6, the relaxivity increase corresponds to complex protonation followed by dissociation, to reach *r*_1_ = 6.7 mM^–1^ s^–1^, characteristic of free [Mn(H_2_O)_6_]^+^.^46^ Between pH 7 and 11, **MnL2** exhibits constant relaxivity (2.62 mM^–1^ s^–1^, at 1.41 T and 25 °C). However, we observe a slight relaxivity decrease for **MnL2** from pH 9 to 10 and for **MnL3** from 7.5 to 9, respectively, as a consequence of partial hydrolysis and the formation of monohydroxo species.

### Dissociation Kinetics

The capacity of Mn(II) complexes to resist dissociation or transmetalation by endogenous ions, e.g., Zn(II) or Cu(II) represents another important parameter for safe *in vivo* use. To describe the kinetic inertness of **MnL2** and **MnL3**, we have evaluated the rate of transmetalation in the presence of Zn(II) and Cu(II) (only for **MnL2**) two relevant endogenous cations, which form more stable complexes than Mn(II) and drive dissociation.

**Figure 4:**
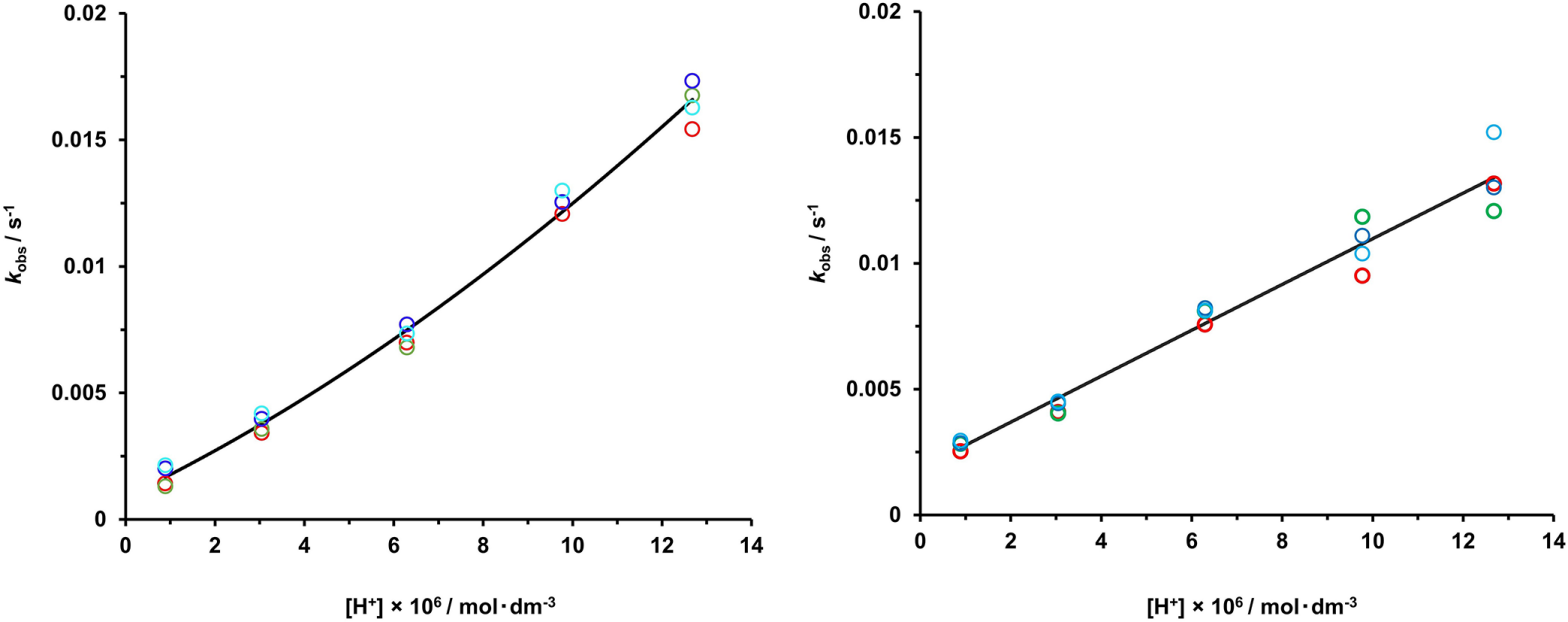
Dependence of the observed dissociation rate constants of **MnL2** (1.4 mM; *left*) and **MnL3** (1.4 mM; *right*) on the proton concentration at 10 (red), 20 (green), 30 (blue), and 40 (light blue) molar equivalents of Zn^2+^. The solid lines correspond to the best fit of the experimental data to eq 3, yielding the parameters in Table 5; *k*_2_ was fixed to zero for **MnL3**.

The dissociation kinetics was followed through monitoring Mn(II) release by water proton *T*_2_ measurements, at pH 4.9–6.1, in the presence of 10- to 40-fold excess of Zn(II) and through UV–vis measurements in the presence of 10- to 40-fold excess of Cu(II) at pH 4.7–5.0 (only for **MnL2**). The observed dissociation rate constants, *k*_obs_, increase with increasing H^+^ ion concentration (Figures 4, *left* and S20). They remain independent of Zn(II) concentration (Figures S18 and S19), but do show dependence on Cu(II) concentration (Figure S21).

**Figure 5:**
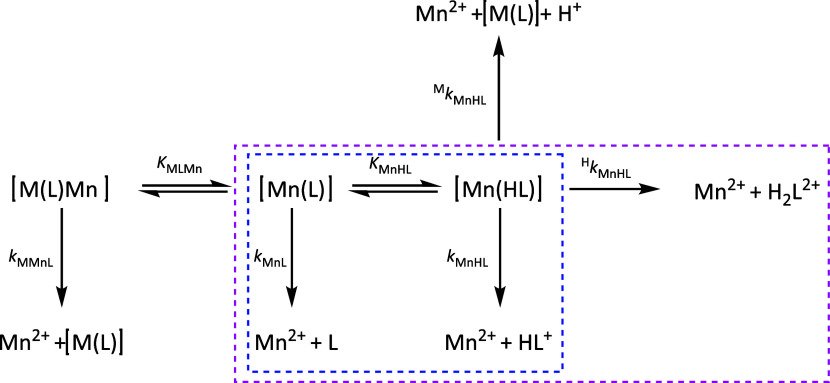
Possible dissociation pathways of **MnL2** and **MnL3**. The pathways having a real contribution to the overall dissociation, as indicated by the fit of the observed rate constants, are highlighted by magenta (**MnL2**) and blue (**MnL3**) dashed lines.

In excess of the exchanging metal ion, the reaction is of pseudo-first order, and the rate is proportional to the total **MnL2** or **MnL3** concentration (eq 1), where *k*_obs_ is the pseudo-first-order rate constant.
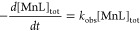
1In general, different dissociation pathways can coexist, implying the participation of protons and/or the exchanging metal ion,^47^ as illustrated in Figure 5.

Based on this scheme, the overall rate for either Zn(II) or Cu(II) transmetalation can be given by eq 2, where M = Zn^2+^ or Cu^2+^ (for clarity, charges of the complexes are omitted).
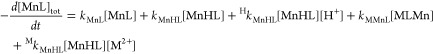
2Here, the first and second terms correspond to the spontaneous dissociation of the nonprotonated and the monoprotonated complexes, respectively, the third term describes the proton-assisted dissociation of the monoprotonated complex, and the last two terms refer to the spontaneous dissociation of the dinuclear complex and metal-assisted dissociation of the monoprotonated **MnL2** or **MnL3** complex. [MnL]_tot_ describes the total concentration of Mn(II) chelates ([MnL]_tot_ = [MnL] + [MnHL] + [ZnLMn]). Since no obvious Zn(II) dependence (for any of the two systems) was observed, only the spontaneous and proton-assisted dissociation pathways were taken into consideration. Consequently, the observed rate constants can be expressed as in eq 3 with *k*_0_ = *k*_MnL_, *k*_1_ = *k*_MnHL_·*K*_MnHL_, and *k*_2_ = ^H^*k*_MnHL_·*K*_MnHL_.
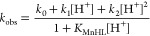
3The *k*_obs_ rate constants were fitted to eq 3, and the calculated parameters are listed in Table 5 for the Zn(II) exchange. During the fitting procedure, *K*_MnHL_ was fixed to the value obtained from the pH-potentiometric titrations.

**Table 5:** Rate and Equilibrium Constants and Dissociation Half-Lives of Selected Mn(II) Complexes

	*k*_0_ (s^–1^)	*k*_1_ (M^–1^ s^–1^)	*k*_2_ (M^–2^ s^–1^)	*k*_3_ (M^–1^ s^–1^)	*k*_4_ (M^–2^ s^–1^)	log *K*_MnHL_^a^	*t*_1/2_ (h)^i^	metal used in transmetalation
L1^b^	(4.1 ± 0.3) × 10^–3^	1691 ± 41				3.82	2.8 min	Zn(II)
**L2**	(1.1 ± 0.3) × 10^–3^	737 ± 110	(4.5 ± 0.8) × 10^7^			3.70	10.4 min	Zn(II)
**L3**	(1.9 ± 0.3) × 10^–3^	912 ± 41	j			2.73	6.1 min	Zn(II)
15-pyN_5_^c^		423	1.0 × 10^7^		1.7 × 10^4^	4.27	11.0	Zn(II)
3,9-PC2A^d^		221 ± 5		(3.6 ± 0.7) × 10^–2^		2.14	21.0	Cu(II)
3,6-PC2A^d^		70 ± 1	(1.5 ± 0.4) × 10^5^	(2.6 ± 0.7) × 10^–2^		3.06	63.2	Cu(II)
PC2A-EA^e^		0.6				6.88	8.00 × 10^3^	Cu(II)
PC2A-BP^f^		16.9 ± 0.2					286.2	Cu(II)
3,9-OPC2A^g^	(8.6 ± 1.1) × 10^–6^	2.81 ± 0.07				(2.40)	21.9	Cu(II)
PC1A^h^		2020 ± 40	(8.0 ± 0.3) × 10^7^			4.95	2.4	Zn(II)

a Obtained by pH-potentiometric titration and fixed during the calculations.

b Ref (30).

c Ref (28).

d Ref (21).

e Ref (22).

f Ref (23).

g Ref (26).

h Ref (20).

i *t*_1/2_ = ln 2/*k*_obs_ (calculated for pH 7.4).

j Fixed to zero during the fitting procedure.

The *k*_0_ values calculated, (1.1 ± 0.3) × 10^–3^ s^–1^ for **MnL2** and (1.9 ± 0.3) × 10^–3^ s^–1^ for **MnL3**, reflect a significant contribution of the spontaneous pathway to the overall dissociation. The spontaneous dissociation of the monoprotonated complex, characterized by the *k*_1_ values, represents another important pathway. For **MnL3**, *k*_2_ was fixed to zero during the fitting procedure, as previously done in the case of **MnL1**; otherwise, very low values were obtained with large errors, indicating that the proton-catalyzed dissociation of the monoprotonated complex is negligible in the investigated pH range. However, the proton-catalyzed dissociation of the monoprotonated complex plays a role in the overall dissociation of **MnL2** which is reflected in *k*_2_ = (4.5 ± 0.8) × 10^7^ M^–2^ s^–1^. A significant contribution of this pathway is manifested in the nonlinear dependency of *k*_obs_ on H^+^ ion concentration (Figure 4, *right*, in contrast to the linear dependency for **MnL3**). We should note that the pH range (4.9–6.1) is relatively limited, which may lead to higher errors on the calculated rate constants; however, below pH 4.9, the dissociation becomes too fast to be followed by conventional techniques. Concerning the Cu(II) exchange, the accessible pH range was even more limited by potential Cu(II) hydrolysis at higher pH. As a consequence, the fitted values for *k*_0_, *k*_1_, and *k*_2_ were obtained with very large errors (Table S3); nevertheless, they are in the same order of magnitude as those calculated from the Zn(II) transmetalation experiment. Here a contribution from the dissociation of the dinuclear Cu(**L2**)Mn species had to be also included in the analysis because of decreasing *k*_obs_ values for increasing Cu(II) concentrations (Figure S21).^47^

As was previously observed for **MnL1**, the introduction of the pendant arm results in slower dissociation as compared to the parent 15-pyN_3_O_2_ macrocycle (which dissociated too fast for relaxometric monitoring). Likewise, very fast dissociation was reported for Mn(15-pyN_3_O_2_Ph).^29^ In the case of **MnL2** and **MnL3**, the process is still fast as compared to the Mn(15-pyN_5_) analogue, which has slower proton-assisted and negligible spontaneous dissociation.^28^ In the family of pyclen derivative Mn(II) complexes, *k*_0_ could not be assessed either (it had to be fixed to zero during the fitting procedures); the only exception is Mn(3,9-OPC2A) where *k*_0_ = (8.6 ± 1.1) × 10^–6^ s^–1^ was reported.^26^ Although the exact reasons are difficult to identify, these data point to the higher relevance of spontaneous dissociation in the case of oxygen-containing macrocycles.

Based on these rate constants, the dissociation half-lives of **MnL2** and **MnL3** were estimated at pH 7.4 and compared to those of other complexes (Table 5). The short dissociation half-lives (in the range of few minutes) are related to the significant contribution of spontaneous dissociation at physiological pH; nevertheless, they are ∼2 to 4 times longer in comparison with **MnL1**. The presence of Mn-ether O bonds in the family of these 15-membered macrocycles clearly disfavors kinetic inertness, as it was already observed for Mn(15-pyN_3_O_2_)^28^ vs Mn(15-pyN_5_). Interestingly, the situation is slightly different for the more rigid 12-membered macrocycles where, for instance, the acid-assisted dissociation is 2 orders of magnitude slower for Mn(3,9-OPC2A) than for the all-N-containing Mn(3,9-PC2A) analogue, even if their overall dissociation half-lives remain comparable at pH 7.4.^21,26^ It is worth pointing out that the negative impact of the oxygen donors in the macrocyclic scaffold is partially compensated for by the beneficial effect of the additional pendant arm on the kinetic inertness. Nevertheless, the modest kinetic inertness of the family of Mn(II) complexes with **L1**-**L3** prevents further *in vivo* investigation in animals.

### ^17^O NMR and ^1^H NMRD

The nuclear relaxation enhancement effect of paramagnetic metal ions is described by the Solomon–Bloembergen–Morgan theory of paramagnetic relaxation.^3^ According to this model, relaxivity is related to the dynamic and structural parameters of the metal complexes. The ^1^H NMRD profiles represent the magnetic field dependency of relaxivity, and their analysis allows for determining microscopic parameters that govern relaxivity. ^1^H NMRD profiles are often complemented by ^17^O variable-temperature NMR measurements providing direct access to the water exchange process, which makes the relaxivity analysis more reliable.

**Figure 6:**
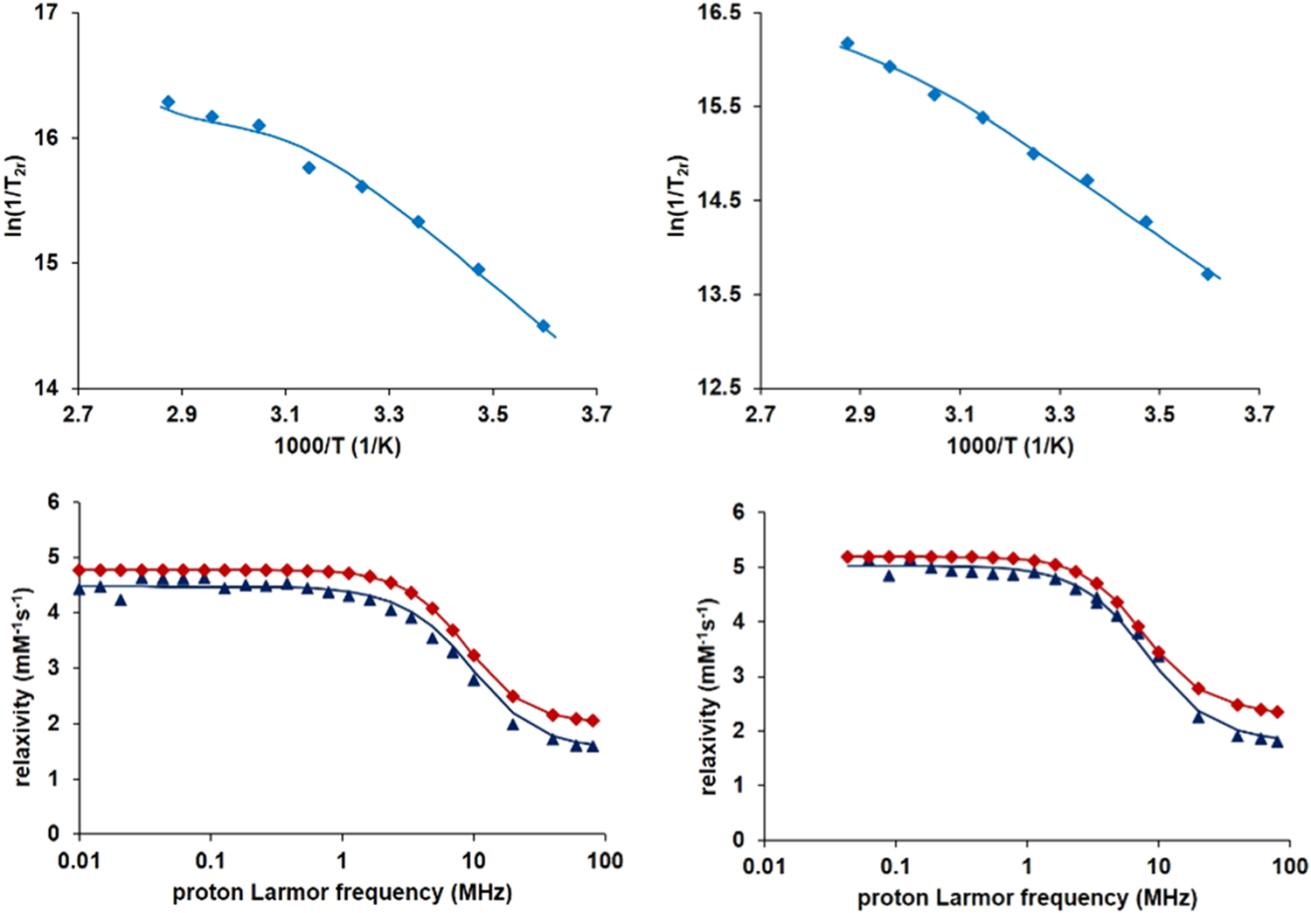
Top: Temperature dependence of the reduced ^17^O transverse relaxation rate for **MnL2** (l*eft*) and **MnL3** (*right*) at 9.4 T and pH 7.4. Bottom: ^1^H NMRD profiles at 25 (*red*) and 37 °C (*blue*); *c*_**MnL2**_ = 0.98 mM at pH = 7.22; and *c*_**MnL3**_ = 0.96 mM at pH = 7.20. Full lines represent the best simultaneous fit of the ^17^O NMR and ^1^H NMRD data.

Variable-temperature transverse ^17^O relaxation times were measured for both **MnL2** and **MnL3** samples in aqueous solution at pH 7.4 where the complexation is complete (Figure 6, *top*). The longitudinal ^17^O relaxation rates were also measured, but due to large errors, the data were not further analyzed. The rate, *k*_ex_^298^, the activation enthalpy, Δ*H*^‡^, and the entropy, Δ*S*^‡^, of the water exchange can be determined from the temperature dependence of the ^17^O transverse relaxation rates, which are dominated by the scalar mechanism.^48^ In the fast exchange regime, where 1/*T*_2r_ decreases with an increase in temperature, transverse ^17^O relaxation rates depend on water exchange, electronic relaxation, and the hyperfine coupling constant *A*_0_/ℏ. However, in the slow exchange regime, where the 1/*T*_2r_ values increase with an increase in temperature, only water exchange is important; this regime is observable only for **MnL2** and **MnL3**.

^1^H NMRD profiles were recorded at 25 and 37 °C in the magnetic field range 0.01–80 MHz (Figure 6, *bottom*). The reduced transverse ^17^O relaxation rates were simultaneously fitted with ^1^H NMRD data according to the Solomon–Bloembergen–Morgan theory (equations are given in SI). The fitted parameters are summarized in Table 6 and compared to those for seven-coordinate Mn(II) complexes of **L1** and 15-pyN_3_O_2_ ligands, as well as of [Mn(H_2_O)_6_]^2+^.

**Table 6:** Relaxivities and Best-Fit Parameters Obtained from the Simultaneous Analysis of ^17^O NMR and ^1^H NMRD Data for **MnL2** and **MnL3** Compared with Those for Other Mn(II) Chelates with Selected 15-Membered Ligands and the Hexaaqua Mn(II) Ion

parameter^f^	[Mn(L1)(H_2_O)]^+^^a^	[Mn(**L2**)(H_2_O)]^+^	[Mn(**L3**)(H_2_O)]^+^	[Mn(15-pyN_3_O_2_)(H_2_O)_2_]^2+^^b^	[Mn(15-pyN_3_O_2_Ph)(H_2_O)_2_]^2+^^c^	[Mn(H_2_O)_6_]^2+^^d^
CN/*q*	7/1	7/1	7/1	7/2	7/2	
*r*_1_ at 25/37 °C (20 MHz)/mM^–1^ s^–1^ at 0.47 T	2.45/2.08	2.49/1.98	2.77/2.24	4.48/3.61	5.16/–	8.36/–
*k*_ex_^298^/10^7^ s^–1^	0.45 ± 0.02	0.46 ± 0.02	0.23 ± 0.02	0.38	0.64	2.1
Δ*H*^‡^/kJ mol^–1^	28.1 ± 2.1	28.7 ± 1.36	28.5 ± 2.5	35.3	34.0	32.9
Δ*S*^‡^/J mol^–1^K^–1^	–23 ± 6	–21 ± 5	–27 ± 7	–1.0	–1.0	+5.7
*E*_rH_/kJ mol^–1^	15.0 ± 0.5	15.0 ± 0.5	15.0 ± 0.5	16.1		
τ_rH_^298^/ps	35 ± 5	35 ± 4	45 ± 2	40.3		30
τ_v_^298^/ps	8 ± 1	8 ± 1	13 ± 3	3.3		3.3
Δ^2^/10^18^ s^–2^	160 ± 10	164 ± 10	100 ± 10	6.6		5.6
*A*_O_/ℏ/10^6^ rad s^–1^	38.6	38.6^e^	38.6^e^	38.6	43	33.3

a Ref (30).

b Ref (28).

c Ref (29).

d Ref (54).

e Fixed during the fitting procedure (the value of the scalar coupling constant in MHz is 6.14).

f *r*_1_: Relaxivity, *k*_ex_^298^: water exchange rate, Δ*H*^‡^: activation enthalpy for the water exchange, Δ*S*^‡^: activation entropy for the water exchange, *E*_*r*H_: activation energy for the rotational motion of the complex, τ_*RH*_: rotational correlation time of the Mn^2+^–H_water_ vector, τ_v_^298^: correlation time for the modulation of the zero-field splitting (ZFS), Δ^2^: trace of the square of the transient ZFS tensor, *A*_O_/*ℏ*: scalar coupling constant.

The shape of the ^1^H NMRD curves reflects typical low-molecular-weight complexes. The profiles display one dispersion between 1 and 10 MHz as has been previously described for other complexes except [Mn(H_2_O)_6_]^2+^.^49,50^ The hydration number of **MnL2** and **MnL3** was assumed in both cases to be *q* = 1 for a total coordination number of CN = 7, based on the X-ray crystal structure. In aqueous solution, the chloride or the acetonitrile that coordinates to Mn(II) in the solid state will be replaced by one water molecule to complete the coordination sphere of Mn(II). As it was described by Gale et al.,^51^ the ^17^O *T*_2_ values can also give an estimate of hydration number, provided the 1/*T*_2r_ vs temperature curve has a maximum. This approach is not applicable for **MnL2** and **MnL3** since both systems are in the slow exchange regime in the entire temperature range studied. The lack of a fast exchange regime prevents the fit of the scalar coupling constant, *A*_0_/ℏ, as well which was therefore fixed to 38.6 MHz.^28^

The relaxivities of our novel monosubstituted 15-pyN_3_O_2_ Mn(II) complexes support *q* = 1 and are comparable to those of **MnL1** or other small monohydrated Mn(II) complexes (Table 7). As expected, they are lower than the *r*_1_ of the bishydrated Mn(15-pyN_3_O_2_) and Mn(15-pyN_3_O_2_Ph) analogues (Table 6). The slightly higher relaxivity observed for **MnL3** vs **MnL2** is likely the result of the larger benzimidazole pendant arm (Table 6) manifested in a slightly higher rotational correlation time, τ_rH_^298^. According to a semiempirical method which was proposed for the determination of the hydration number for Mn(II) complexes and which is based on the molecular weight of the chelate and the low field relaxivity (0.01 MHz, if the NMRD profile has one dispersion),^52^ we estimate *q* = 0.73 (**MnL2**) and *q* = 0.76 (**MnL3**), which are in reasonable accordance with the assumed monohydration.

**Table 7:** Comparison of Relevant Physicochemical Data of the Mn(II) Complexes Formed with **L2, L3**, and Other Selected Ligands

	[Mn(**L2**)(H_2_O)]^+^	[Mn(**L3**)(H_2_O)]^+^	[Mn(15-pyN_5_)(H_2_O)_2_]^2+^^a^	[Mn(3,9-PC2A)(H_2_O)]^b^	[Mn(3,6-PC2A)(H_2_O)]^b^	[Mn(PC2A-EA)(H_2_O)]^c^
CN/*q*	7/1	7/1	7/2	7/1	7/1	7/0 7/1^d^
*r*_1p_298^e^/mM^–1^ s^–1^	2.49	2.77	3.56	2.91	2.72	2.1^f^/3.5^g^
*k*_ex_^298^/10^7^ s^–1^	0.46 ± 0.02	0.23 ± 0.02	6.9 ± 0.7	12.6 ± 1.2	14.0 ± 2.5	4.0 ± 0.2
Δ*H*^‡/^/kJ mol^–1^	28.7 ± 1.36	28.5 ± 2.5	37.7 ± 4	37.5 ± 2.4	38.2 ± 3.9	

		[Mn(PC2A-BP)(H_2_O)]^h^	[Mn(EOB-PC2A)(H_2_O)]^i^	[Mn(3,9-OPC2A)(H_2_O)]^j^	[Mn(PC1A)(H_2_O)]^+^^k^	[Mn(PC1P)(H_2_O)]^k^
CN/*q*		7/1	7/1	7/1	6/1	6/1
*r*_1p_298^g^/mM^–1^ s^–1^		4.96/35.7^l^	2.8/5.9 (1.5 T)^m^	3.09	2.39	2.84
*k*_ex_^298^/10^7^ s^–1^		6.3 ± 0.2		5.3 ± 0.4	303 ± 19	177 ± 9
Δ*H*^‡^/kJ mol^–1^		35.7 ± 0.1		28.5 ± 1.7	13.0 ± 1.6	14.0 ± 1.2

a Ref (28).

b Ref (21).

c Ref (22).

d Ref (22); at pH value above 8.0.

e Determined at 298 K and 0.47 T.

f Determined at pH 6.0.

g Determined at pH 7.4.

h Ref (29).

i Ref (24).

j Ref (26).

k Ref (20).

l Ref (55); measured in the presence of HSA.

m Ref (24).

The calculated water exchange rates show little variation in this family of 15-membered macrocyclic complexes, and in overall, they are rather low (*k*_ex_^298^ = 0.23 × 10^7^ s^–1^ for **MnL3** is the lowest value ever reported for a Mn(II) chelate). As we suggested in previous work,^28^ the slow water exchange rate in the whole family of Mn(II) complexes formed with 15-pyN_3_O_2_ derivatives is likely related to the formation of hydrogen bonds between the coordinated water molecules and the ether oxygen donor atoms of the macrocycle. This effect is nonexisting in the complexes of 15-pyN_5_ analogues. The slower water exchange for the benzimidazole derivative as compared to the pyridine analogue might be the consequence of the bulkier pendant arm, which hampers the entry of the incoming water molecule in the inner sphere in an associatively activated water exchange process. Indeed, associative activation is indicated by negative values of the activation entropy. Given the slow water exchange of **MnL2** and **MnL3**, and thus the lack of a fast exchange regime in the ^17^O transverse relaxation curve (Figure 6, *top*), in our analysis, the electronic relaxation parameters are determined by the NMRD data, essentially at low magnetic fields. Differences reflected in higher values of τ_v_^298^ and Δ^2^ might be related to different symmetry; nevertheless, any deeper interpretation is difficult despite some recent efforts to relate structure and electron spin relaxation of Mn(II) complexes.^53^

### Cyclic Voltammetry

The potential oxidation of a Mn(II)-based CA may have a negative impact on its stability, as well as its relaxation efficacy, since relaxivity may be dramatically reduced for the oxidized, low-spin Mn(III).^20,56^ Thus, we carried out CV experiments on an aqueous solution of the Mn(II) complexes in 0.15 M KCl. The cyclic voltammogram of **MnL2** recorded at 100 mV s^–1^ scan rate displays an oxidation peak at *E*_ox_ = +994 mV and a reduction peak at *E*_red_ = +850 mV (*E*_1/2_ = 922 mV vs Ag/AgCl, Δ*E* = 144 mV) (Figure S22). The cyclic voltammogram of **MnL3** is similar, showing an oxidation peak at *E*_ox_ = +1004 mV and a reduction peak at *E*_red_ = 782 mV (*E*_1/2_ = 893 mV vs Ag/AgCl, Δ*E* = 222 mV) (Figure S23). The large separation between the oxidation and reduction peaks (Δ*E*) is characteristic of a quasi-reversible system. The irreversibility of the Mn(II)/Mn(III) redox process is quite common due to the lack of ligand-field stabilization for the high-spin *d*^5^ configuration of Mn(II), with a consequent large inner-sphere contribution to electron transfer.^57^ Even larger separation between the oxidation and reduction peaks was observed when the complexes were measured in acetonitrile (Figures S24 and S25 in SI).

The *E*_ox_ values determined for **MnL2** and **MnL3** are shifted to higher potentials as compared to Mn(3,6-PC2A) (*E*_ox_ = +862 mV), Mn(3,9-PC2A) (*E*_ox_ = +838 mV) or Mn(EDTA) (*E*_ox_ = +769 mV) measured under similar conditions in 0.15 NaCl and 2 mM concentration.^21,58^ This is in accordance with the stronger π-acceptor ability of pyridine and benzimidazole moieties versus the carboxylate group, which is considered rather as an π-donor, resulting in stronger stabilization of the lower oxidation state Mn(II). Similar behavior has been previously observed for complexes with disubstituted derivatives of 15-pyN_3_O_2_ containing two carboxylate, pyridine, or benzimidazole pendant arms.^33,59^ Moreover, pyridine was found to be a better π-acceptor than benzimidazole which has been manifested by higher redox potential,^59^ as in our case (*E*_1/2_ = 922 mV for **MnL2** is larger than *E*_1/2_ = 893 mV for **MnL3)**. A similar value has been also reported for the nonsubstituted Mn(15-pyN_3_O_2_) (*E*_ox_ = 912 mV).^28^ In overall, the high redox potentials of **MnL2** and **MnL3** provide evidence that these chelators both have similar and very good ability to stabilize the Mn(II) oxidation state.

## Conclusions

With the aim of expanding the family of 15-pyN_3_O_2_-based ligands, we have prepared and characterized two new chelators bearing either a 2-pyridylmethyl (**L2**) or a 2-benzimidazolylmethyl (**L3**) pendant arm. Despite synthetic and purification difficulties, both ligands could be obtained in good yield. The preparation of **L2** involved an additional step including the use of Boc-protecting groups in order to differentiate the retention factors in column chromatography and separate pure product **L2** from the unreacted parent macrocycle 15-pyN_3_O_2_ with a higher yield. The crystal structures of both **MnL2** and **MnL3** complexes confirmed CN = 7 and a pentagonal bipyramidal coordination geometry, whereas the crystal structures of both Cu(II) analogues evidenced a strongly distorted coordination environment. The Mn(II) complexes were characterized for their stability, inertness, and relaxation efficacy. The introduction of the pendant arm in the ligand scaffold led to a higher thermodynamic stability in comparison with the parent 15-pyN_3_O_2_ macrocycle, as also observed for the previously studied **MnL1** chelate with an acetate pendant. While the thermodynamic stability constants are slightly lower for **MnL2** and **MnL3** than for the acetate analogue, their pMn values are comparable. The Mn(II) complexes are fully formed at pH 6; however, hydrolysis is observed at basic pH. Interestingly, the kinetic inertness of these complexes is improved by a factor of 2–4 not only in comparison to the parent 15-pyN_3_O_2_ analogue but also to **MnL1**, resulting from slower dissociation of the protonated species. **MnL2** displays the longest half-live, *t*_1/2_ = 10.4 min, among all Mn(15-pyN_3_O_2_) derivatives. The relaxivities of **MnL2** and **MnL3** are comparable to that of **MnL1** and other small Mn(II) chelates with one inner-sphere water molecule (*r*_1_ = ∼2.5 mM^–1^ s^–1^ at 20 MHz/0.47 T). The water exchange rate showed only a little variation in this 15-membered macrocyclic family, and **MnL3** possesses the lowest water exchange rate ever measured for a Mn(II) chelate (*k*_ex_^298^ = 0.23 × 10^7^ s^–1^). Finally, cyclic voltammetry demonstrated that both ligands have good ability to favor the Mn(II) over the Mn(III) oxidation state. Overall, these results illustrate that in this class of 15-membered pyridine-based macrocyclic Mn(II) chelates, thermodynamic stability and relaxation properties remain relatively independent of the nature of the pending coordinating function (pyridine, benzimidazole, or carboxylate), while the kinetic inertness is slightly enhanced for the pyridine derivative.

## Supporting Information Available

MS, ^1^H, ^13^C, and 2D NMR spectra for ligands **L2** and **L3**; crystal packing of complexes **1** and **2**; molecular structure of Cu(L2-Boc); potentiometric titration curves for **L2** and **L3**; additional dissociation kinetic data; and cyclic voltammograms for **MnL2** and **MnL3** (PDF)
