# Supplementary material for: Expanding the Family of Monosubstituted 15-Membered Pyridine-Based Macrocyclic Ligands for Mn(II) Complexation in the Context of MRI
Source: Inorg Chem. 2025 Apr 11;64(16):8205–21. doi: 10.1021/acs.inorgchem.5c00452 (PMC12042267; doi:10.1021/acs.inorgchem.5c00452)
Supplement: Supplementary file 1 — ic5c00452_si_001.pdf [file ic5c00452_si_001.pdf]

Supporting information

for

Expanding the family of monosubstituted 15-membered pyridine-based macrocyclic ligands for Mn(II) complexation in the context of MRI

Marie Pražáková,[a] Daouda Ndiaye,[b] Éva Tóth*[b] and Bohuslav Drahoš*[a]

[a] Department of Inorganic Chemistry, Faculty of Science, Palacký University Olomouc, 17. listopadu 12, 771 46 Olomouc, Czech Republic, Fax: +420 585 634 954. Tel: +420 585 634 429. E-mail: bohuslav.drahos@upol.cz
b Centre de Biophysique Moléculaire, UPR 4301, CNRS, rue Charles Sadron, 45071 Orléans, France, Fax: +33-23863151. E-mail: eva.jakabtoth@cnrs-orleans.fr

Table of contents:

Figure S1 MS spectrum (+) of studied ligand **L2.**

Figure S2 MS spectrum (+) of studied ligand **L3.**

Figure S3 ^{1}H NMR spectrum of studied ligand **L2.**

Figure S4 ^{13}C NMR spectrum of studied ligand **L2.**

Figure S5 HMQC spectrum of studied ligand **L2.**

Figure S6 HMBC spectrum of studied ligand **L2.**

Figure S7 ^{1}H NMR spectrum of studied ligand **L3.**

Figure S8 ^{13}C NMR spectrum of studied ligand **L3.**

Figure S9 HMQC spectrum of studied ligand **L3.**

Figure S10 HMBC spectrum of studied ligand **L3.**

Figure S11 IR spectrum of the 15-pyN_3O_2 Schiff base(s) reaction mixture.

Figure S12 ^{1}H NMR spectrum of **L2**-Boc.

Figure S13 Crystal packing of complex **1.**

Figure S14 Crystal packing of complex **2.**

Figure S15 Molecular structure of Cu(**L2**-Boc).

Figure S16 Potentiometric titration curves for ligand **L2** in the absence and in the presence of selected divalent metal ions.

Figure S17 Potentiometric titration curves for ligand **L3** in the absence and in the presence of selected divalent metal ions.

Figure S18 Dependence of the observed dissociation rate constants for **MnL2** on Zn^{2+} concentration.

Figure S19 Dependence of the observed dissociation rate constants for **MnL3** on Zn^{2+} concentration.

Figure S20 Dependence of the observed dissociation rate constants for **MnL2** on H^{+} concentration.

Figure S21 Dependence of the observed dissociation rate constants for **MnL2** on Cu^{2+} concentration.

Figure S22 Cyclic voltammogram of **MnL2** in 0.15 M KCl.

Figure S23 Cyclic voltammogram of **MnL3** in 0.15 M KCl.

Figure S24 Cyclic voltammogram of **MnL2** in 0.1 M TBAP in CH_3CN.

Figure S25 Cyclic voltammogram of **MnL3** in 0.1 M TBAP in CH_3CN.

Scheme S1 Reaction scheme describing Schiff bases formation.

Scheme S2 Reaction scheme of preparation, purification and deprotection of **L2**-Boc and Boc_2-15-pyN_3O_2 intermediates leading to the pure product **L2** and recovery of 15-pyN_3O_2.

Table S1 Crystal data and structure refinements for the Cu(II) complex of **L2**-Boc

Table S2 Selected interatomic distances and angles for the Cu(II) complex of **L2**-Boc

Table S3 Fitted rate and equilibrium constants for transmetalation reaction of **MnL2** with Cu(II)

Analysis of the ^{17}O NMR and ^{1}H NMRD data

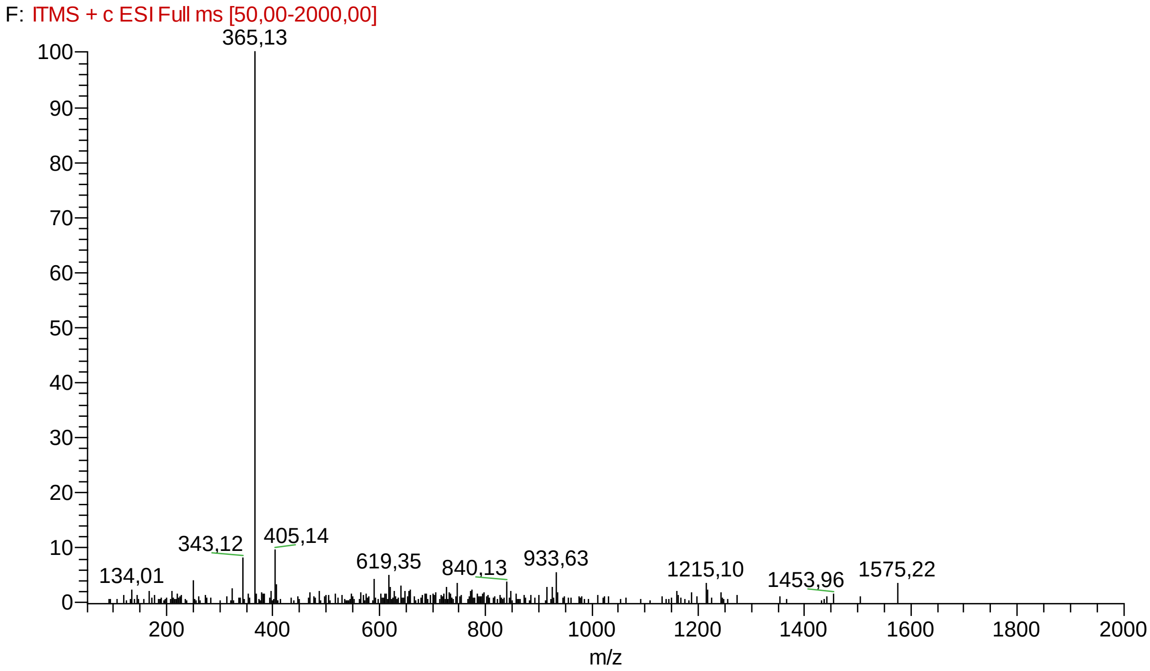

Figure S1 MS (*m/z*) (+) spectrum of studied ligand **L2.**

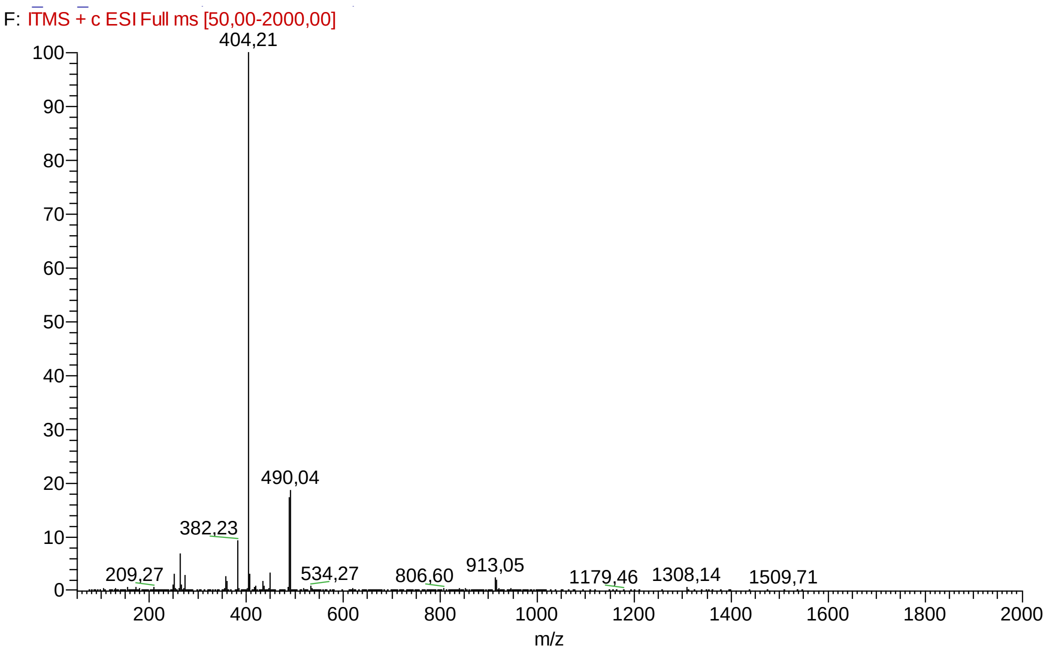

Figure S2 MS (*m/z*) (+) spectrum of studied ligand **L3.**

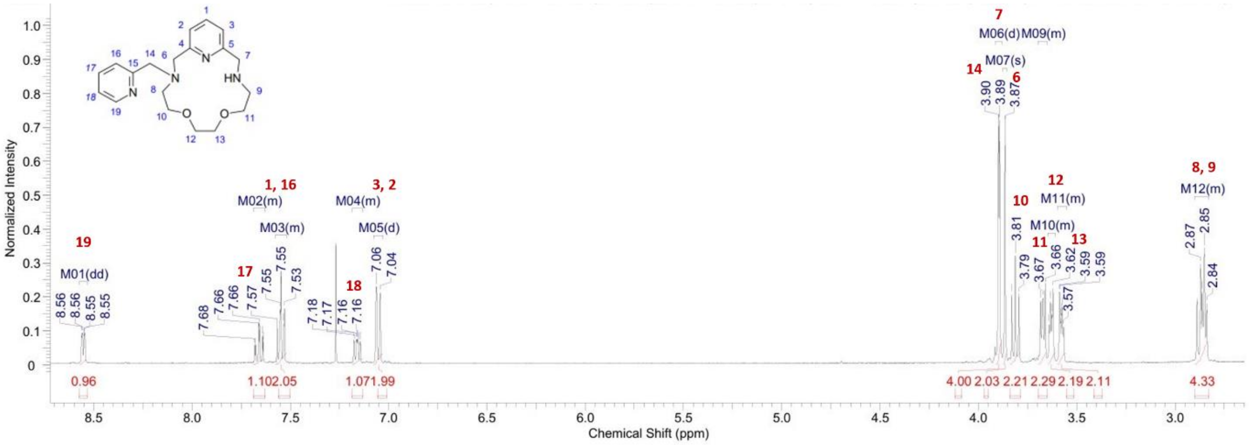

Figure S3 ^{1}H NMR (400 MHz, $CDCl_3$) spectrum of studied ligand **L2.**

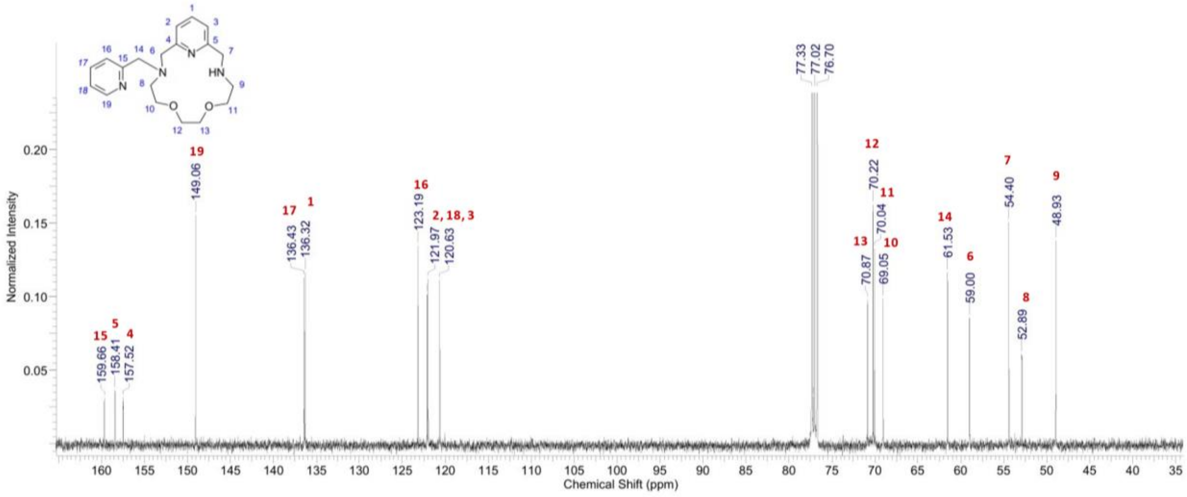

Figure S4 ^{13}C NMR (400 MHz, $CDCl_3$) spectrum of studied ligand **L2.**

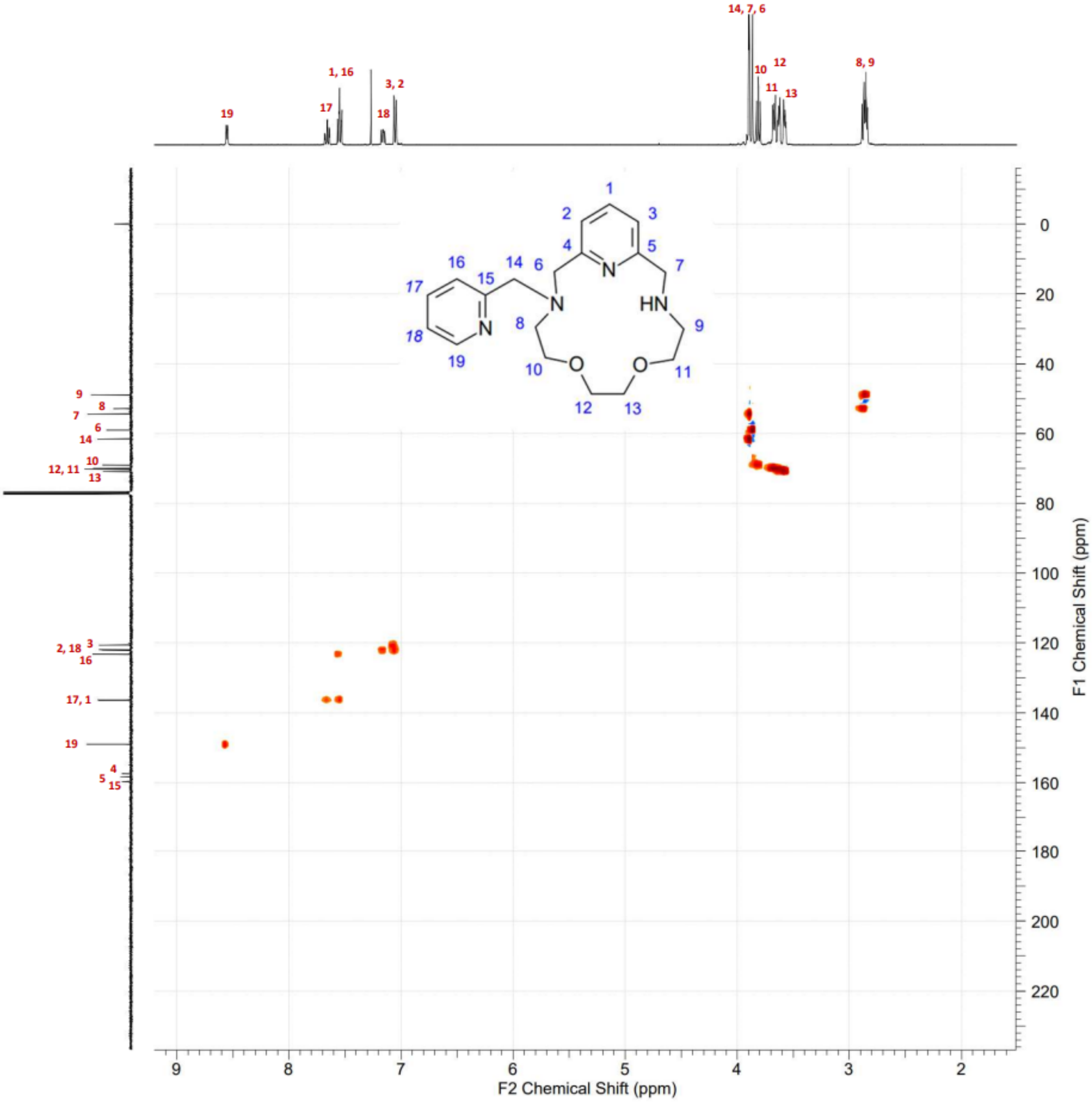

Figure S5 ^{1}H–^{13}C HMQC (400 MHz, $CDCl_3$) spectrum of studied ligand **L2.**

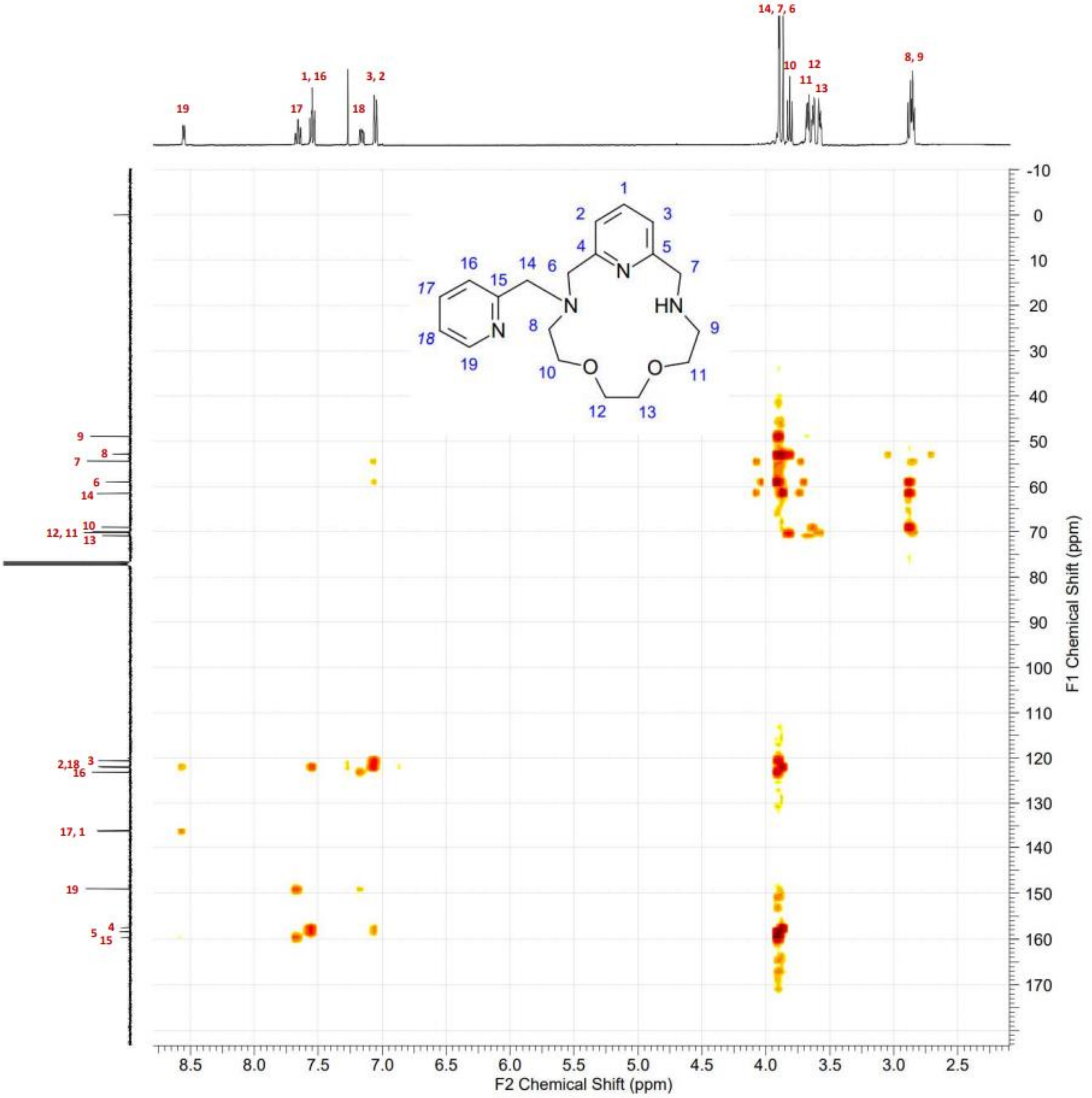

Figure S6 ^{1}H–^{13}C HMBC (400 MHz, $CDCl_3$) spectrum of studied ligand **L2.**

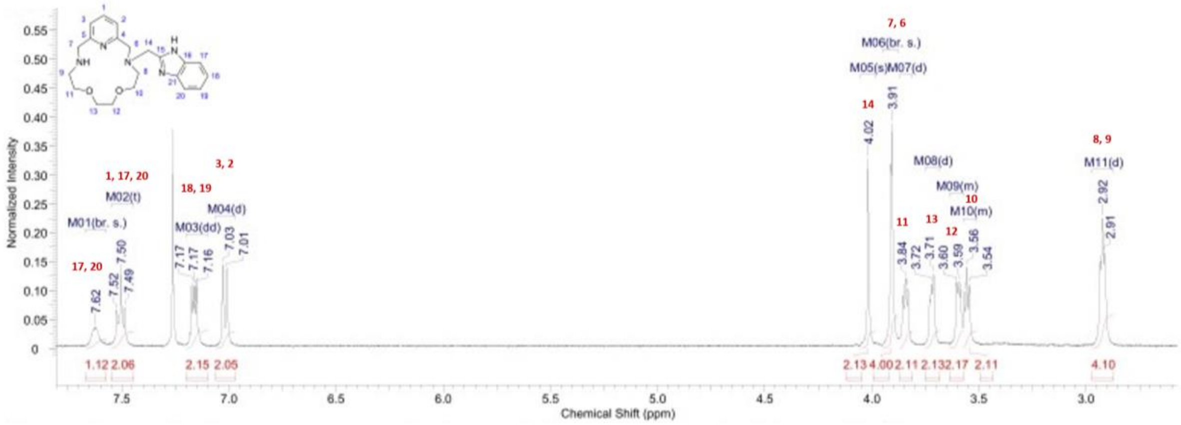

Figure S7 ^{1}H NMR (400 MHz, $CDCl_3$) spectrum of studied ligand **L3.**

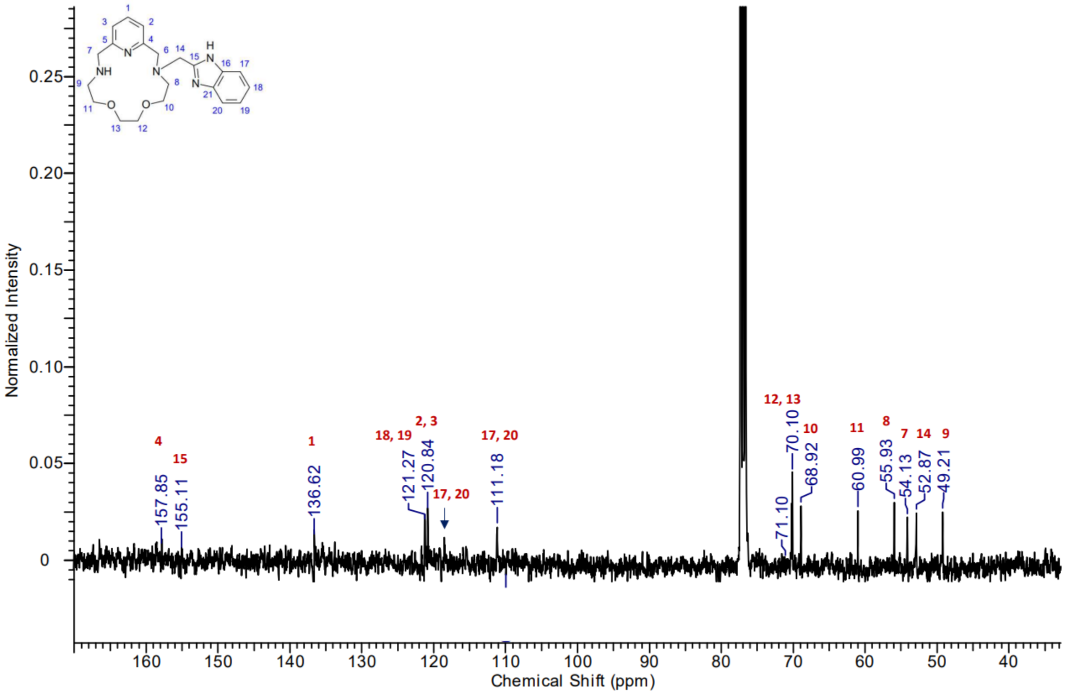

Figure S8 ^{13}C NMR (400 MHz, $CDCl_3$) spectrum of studied ligand **L3.**

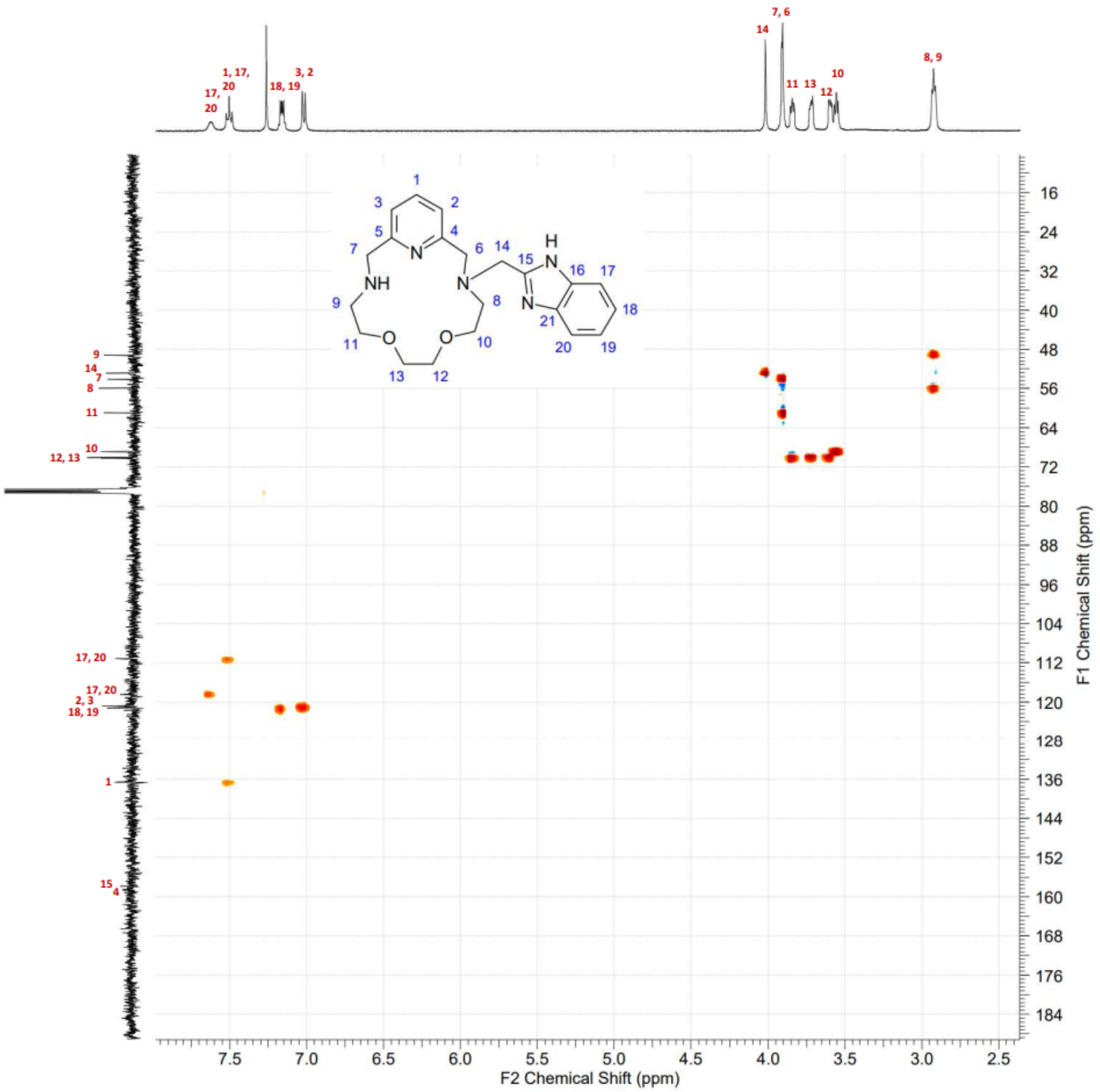

Figure S9 ^{1}H–^{13}C HMQC (400 MHz, $CDCl_3$) spectrum of studied ligand **L3.**

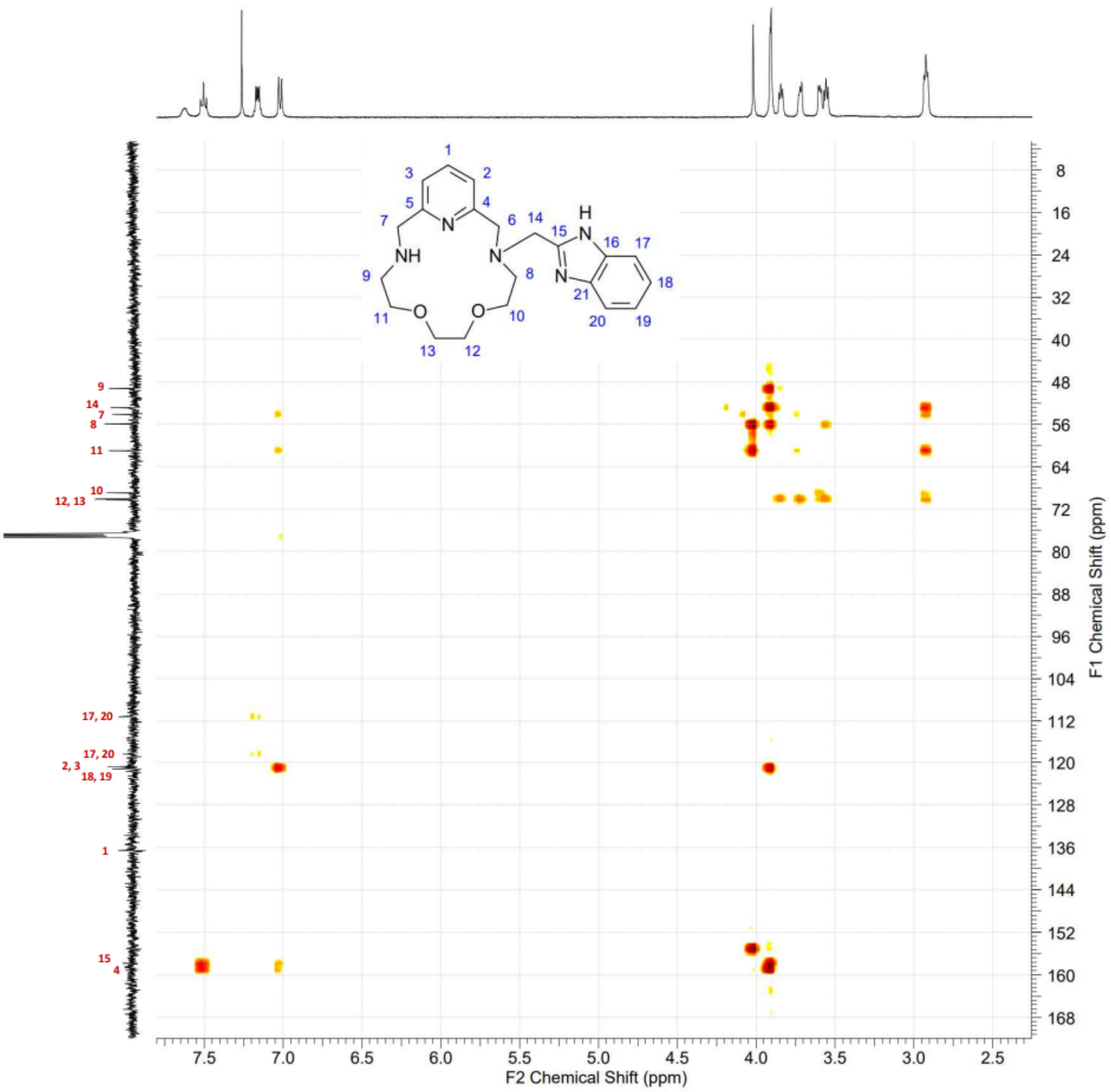

Figure S10 ^{1}H–^{13}C HMBC (400 MHz, $CDCl_3$) spectrum of studied ligand **L3.**

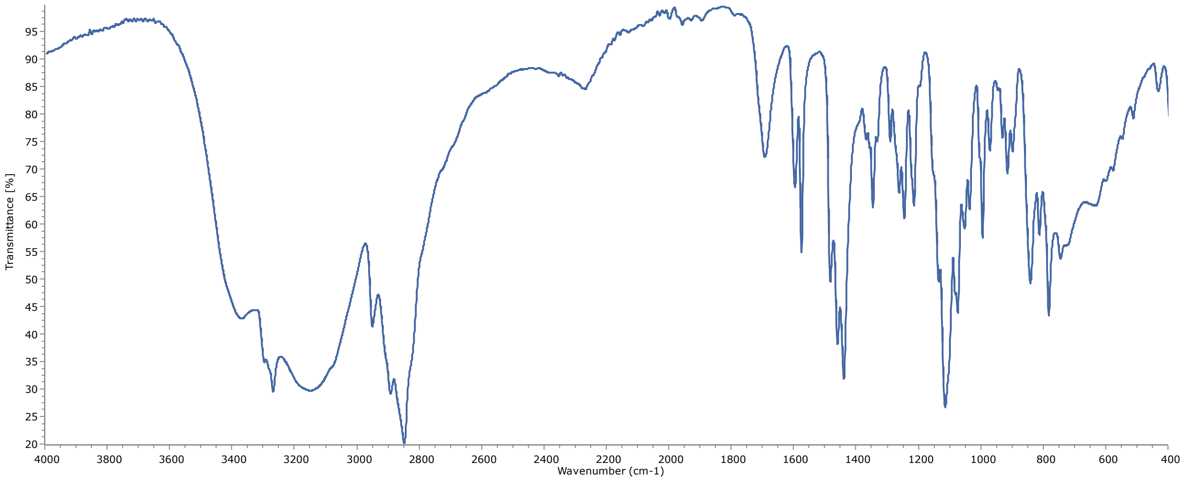

Figure S11 IR spectrum of the 15-pyN_3O_2 Schiff base(s) reaction mixture.

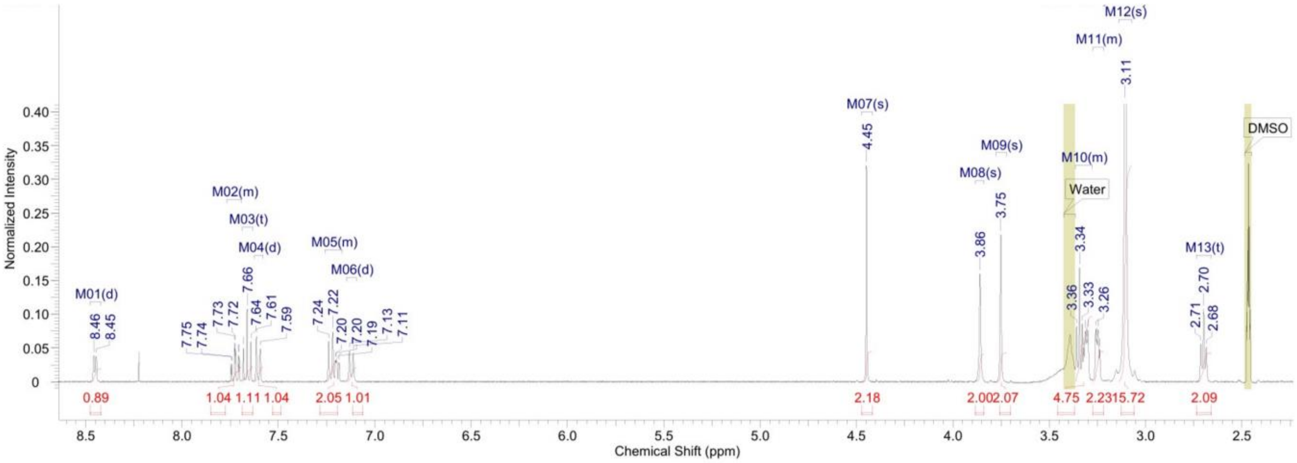

Figure S12 ^{1}H NMR (400 MHz, dmso-d_6; 70 °C) spectrum of **L2**-Boc intermediate.

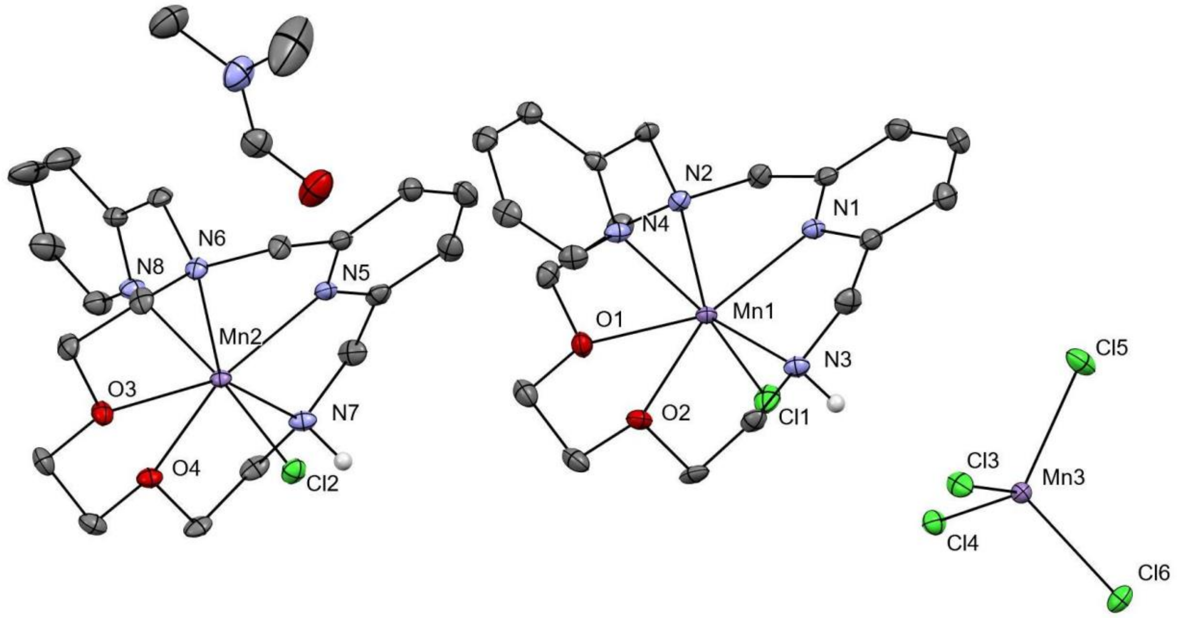

Figure S13 Crystal packing of complex [Mn**L2**Cl]$_2$[MnCl$_4$]·DMF (complex **1**). Hydrogen atoms, except those in NH groups, are omitted for clarity. The thermal ellipsoids are drawn with 50% probability.

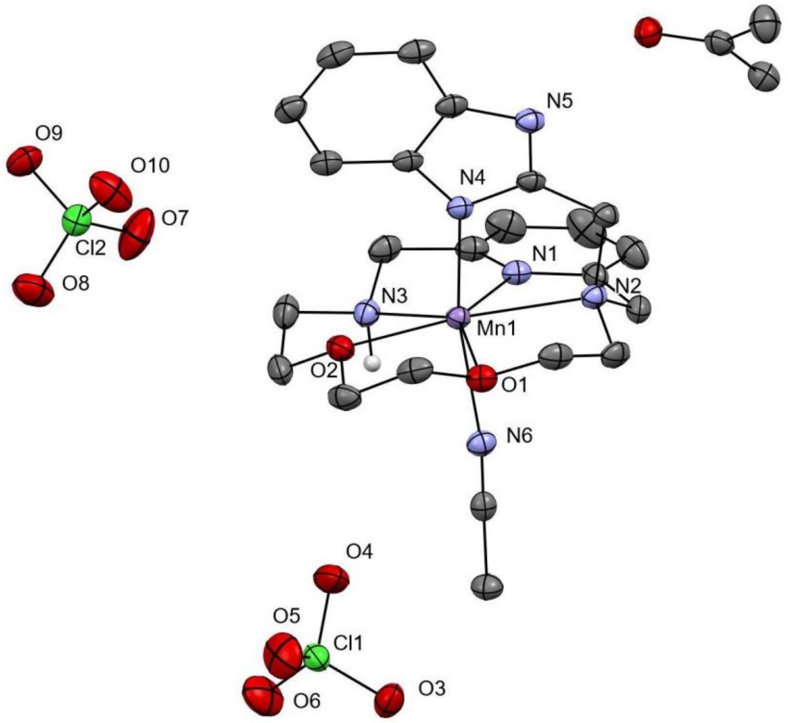

Figure S14 Crystal packing of complex [Mn**L3**](ClO$_4$)$_2$·(CH$_3$)$_2$CO (complex **2**). Hydrogen atoms, except those in NH groups, are omitted for clarity. The thermal ellipsoids are drawn with 50% probability.

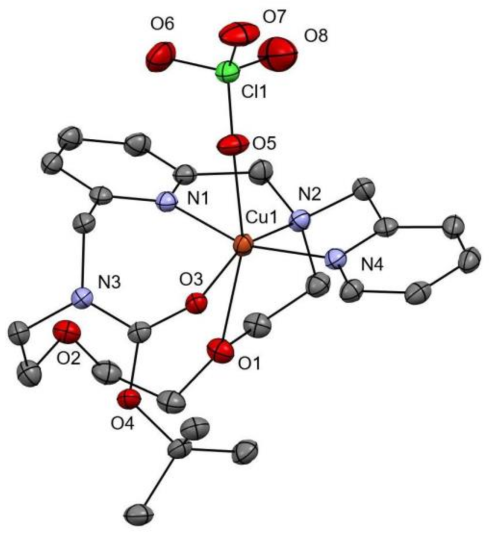

Figure S15 Molecular structure of [Cu(**L2**-Boc)(ClO_4)]$^+$ found in the crystal structure of [Cu(**L2**-Boc)(ClO_4)](ClO_4) CH_3CN. Hydrogen atoms are omitted for clarity. The thermal ellipsoids are drawn at the 50% probability level.

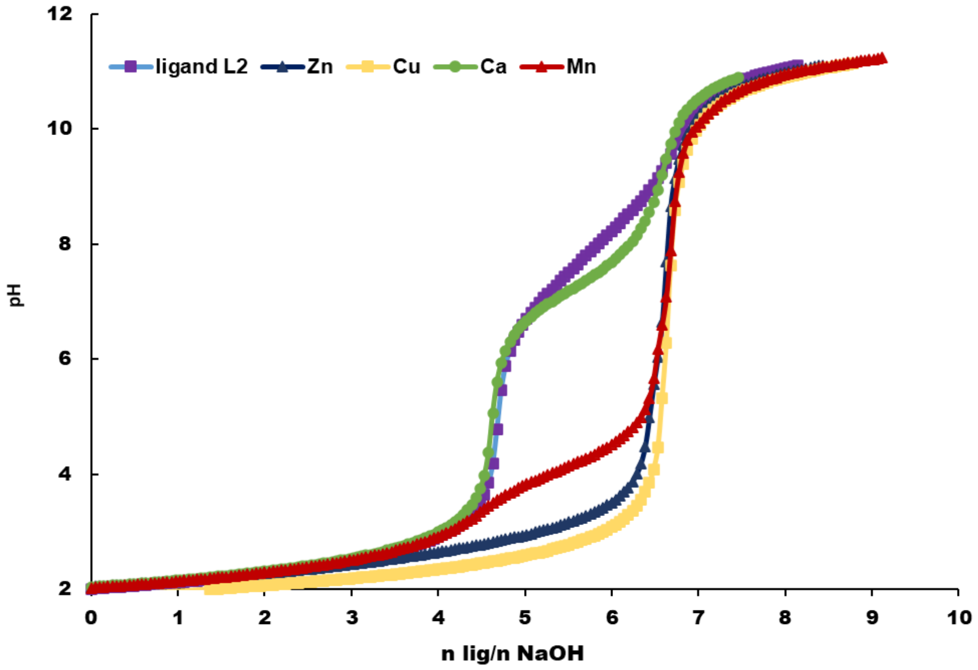

Figure S16 Potentiometric titration curves, as a dependence of pH on *n* lig/ *n* NaOH, obtained for ligand **L2** in the absence and in the presence of selected divalent metal ions (I = 0.15M; 25°C).

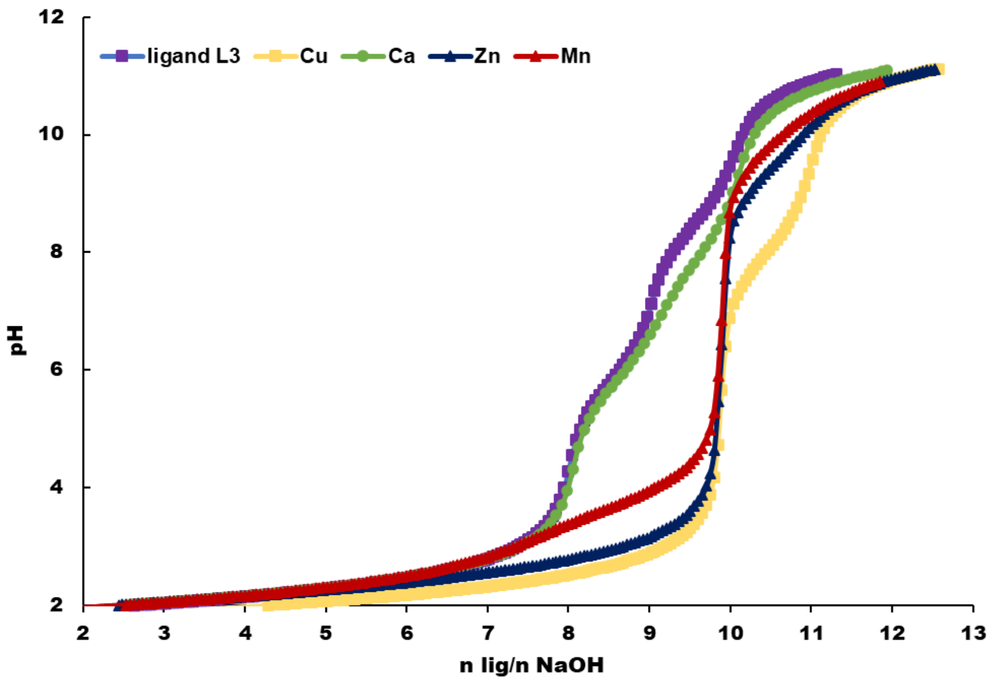

Figure S17 Potentiometric titration curves, as a dependence of pH on *n* lig/ *n* NaOH, obtained for ligand **L3** in the absence and in the presence of selected divalent metal ions (I = 0.15M; 25 °C).

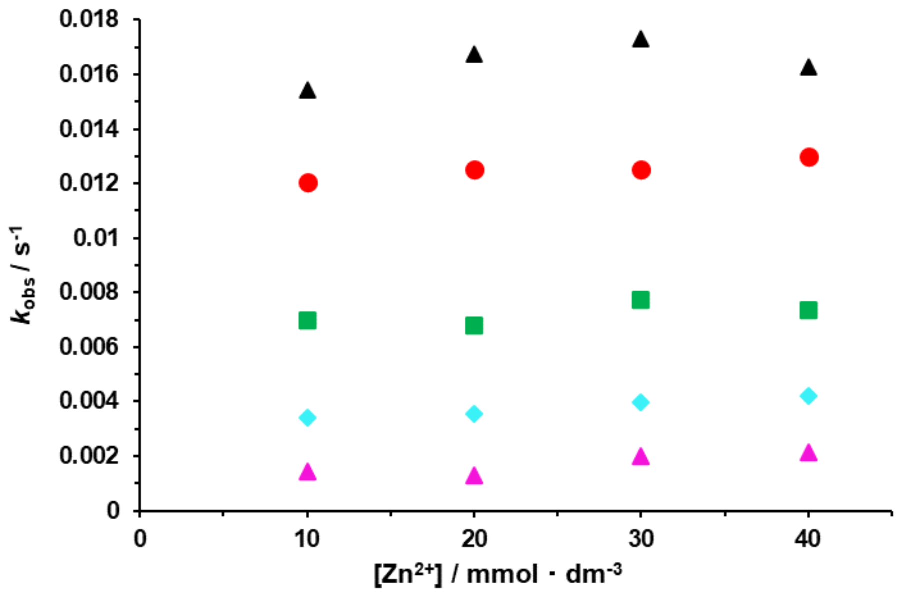

Figure S18 Dependence of the observed dissociation rate constants for **MnL2** on Zn^{2+} concentration. pH readings from the top are 4.9 (*black*), 5.0 (*red*), 5.2 (*green*), 5.5 (*light blue*) and 6.1 (*pink*).

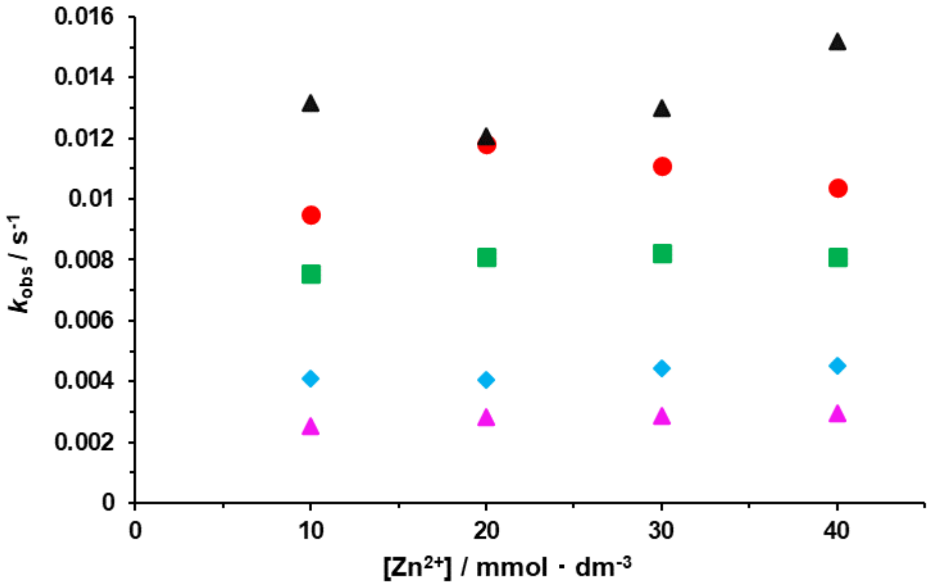

Figure S19 Dependence of the observed dissociation rate constants for **MnL3** on Zn^{2+} concentration. pH readings from the top are 4.9 (*black*), 5.0 (*red*), 5.2 (*green*), 5.5 (*light blue*) and 6.1 (*pink*).

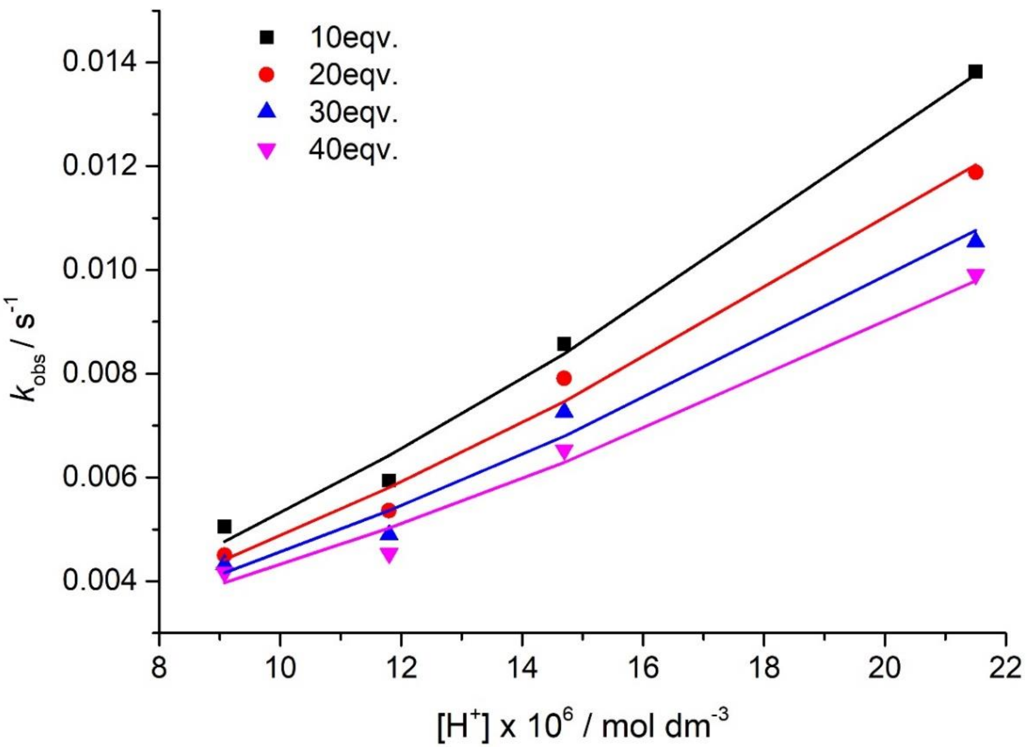

Figure S20 Dependence of the observed dissociation rate constants for **MnL2** (0.3 mM) on pH for different Cu^{2+} concentrations (expressed in equivalents of **MnL2** concentration). The solid lines correspond to the best fit to equation (S1) yielding the parameters given in Table S3.

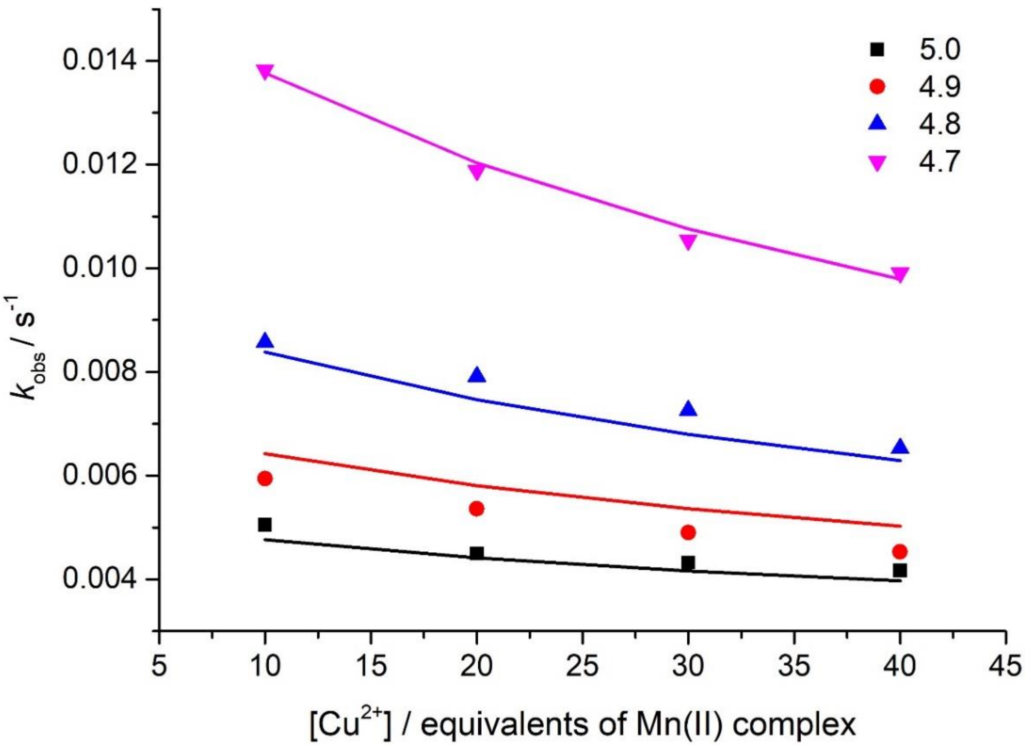

Figure S21 Dependence of the observed dissociation rate constants for **MnL2** (0.3 mM) on Cu^{2+} concentration for different pH values. The solid lines correspond to the best fit to equation (S1) yielding the parameters given in Table S3.

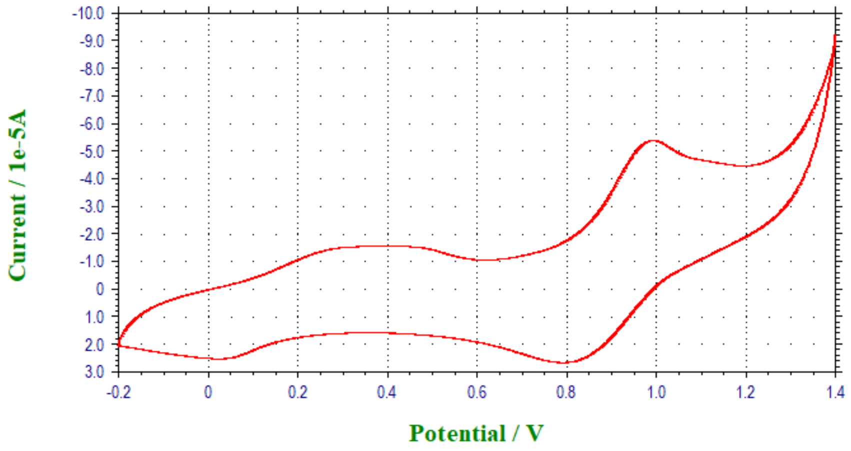

Figure S22 Cyclic voltammogram of 1.5 mM **MnL2** in 0.15 M KCl.

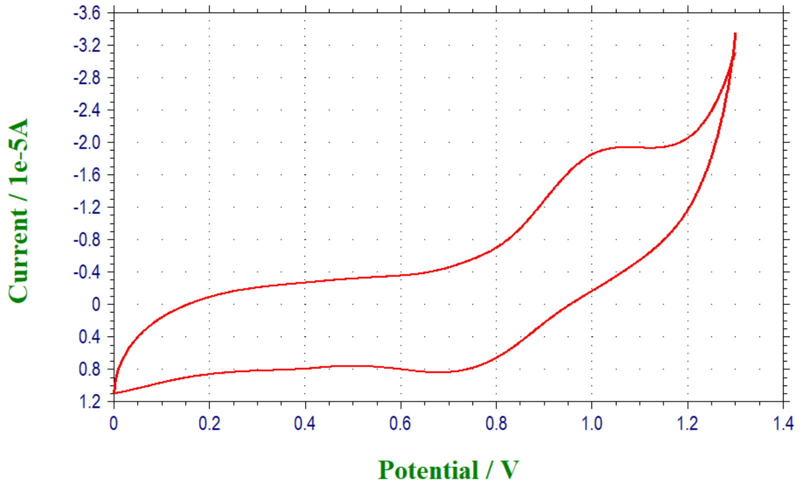

Figure S23 Cyclic voltammogram of 1.5 mM **MnL3** in 0.15 M KCl.

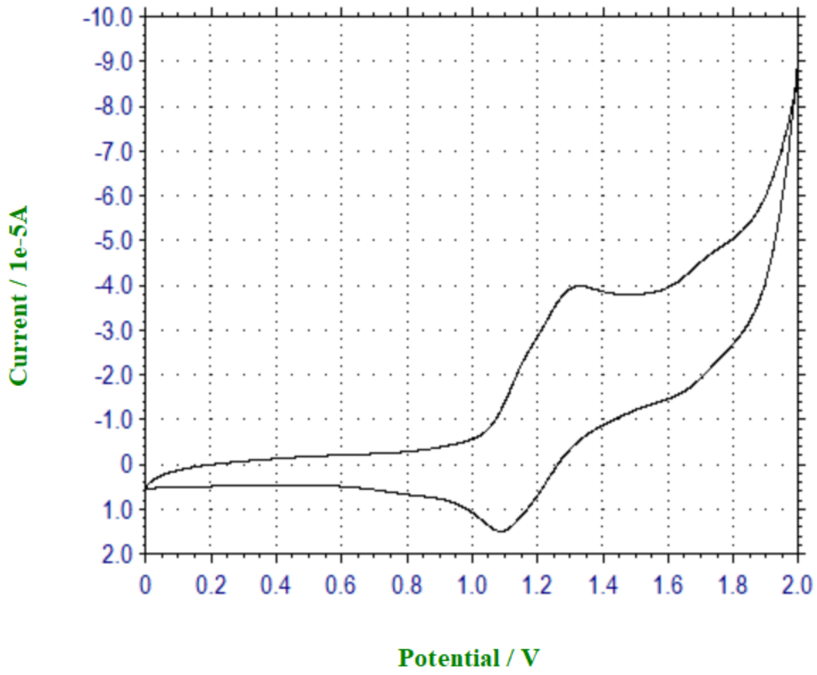

Figure S24 Cyclic voltammogram of 1.5 mM **MnL2** in 0.1 M TBAP in CH_3CN.

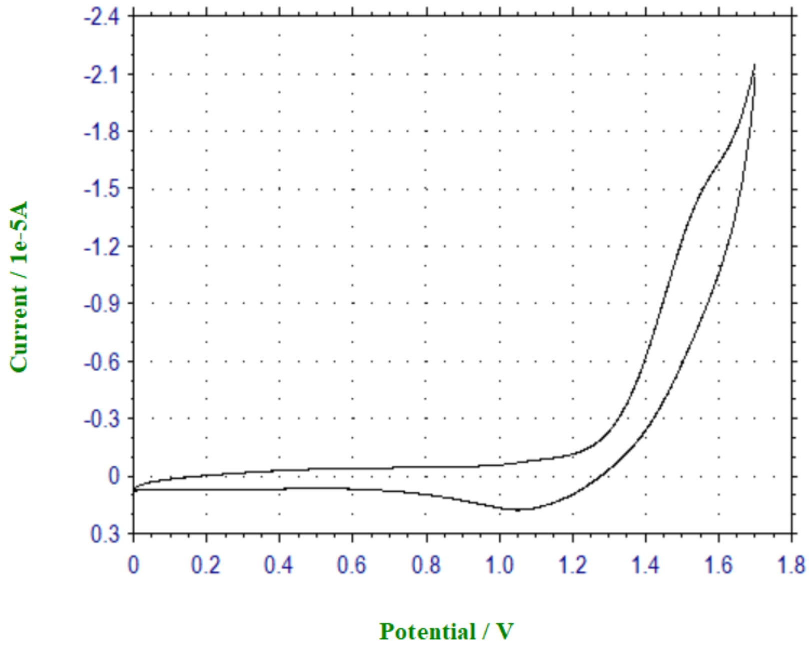

Figure S25 Cyclic voltammogram of 1.5 mM **MnL3** in 0.1 M TBAP in CH_3CN.

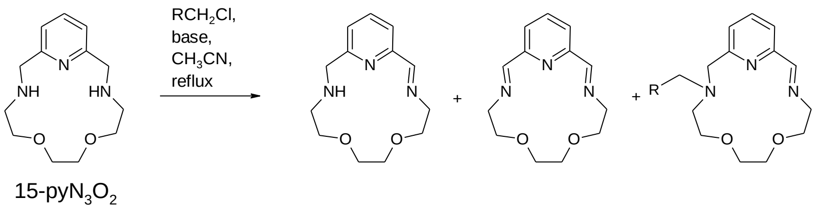

Scheme S1 Reaction scheme describing Schiff bases formation.

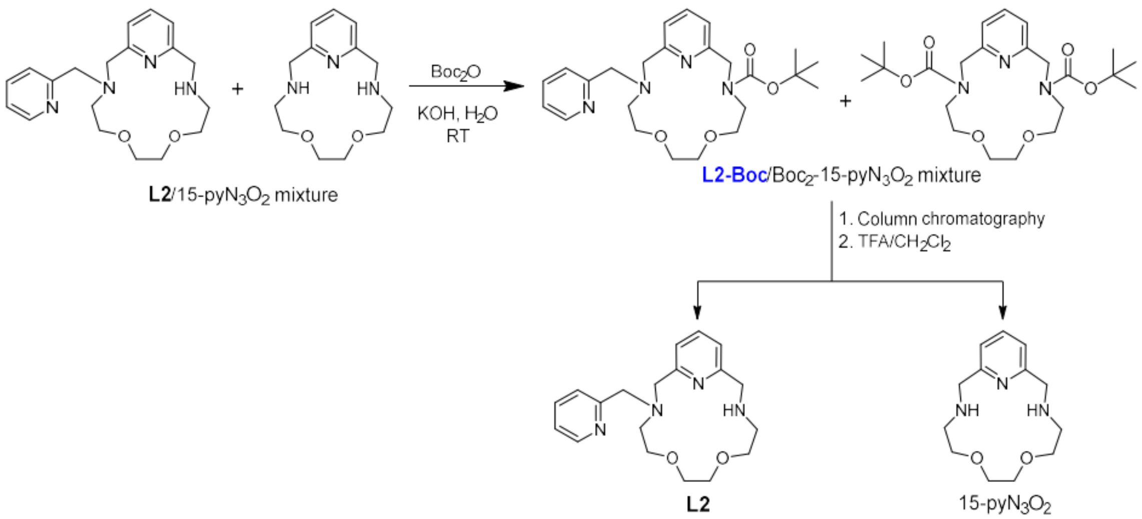

Scheme S2 Reaction scheme of preparation, purification and deprotection of **L2**-Boc and Boc_2-15-pyN_3O_2 intermediates leading to the pure product **L2** and recovery of 15-pyN_3O_2.

Table S1 Crystal data and structure refinements for the Cu(II) complex of **L2**-Boc

Formula	$C_{26}H_{37}Cl_2CuN_5O_{12}$
M_r	746.04
Temperature (K)	100.00(10)
Crystal system	Triclinic
Space group	*P*-1
a (Å)	11.1635(4)
b (Å)	12.6981(4)
c (Å)	12.8778(3)
α (°)	86.784(2)
β (°)	64.893(3)
γ (°)	74.764(3)
V, Å^3	1591.71(9)
Z	2
D_{calc} (g cm^{-3})	1.557
μ (mm^{-1})	3.119
F(000)	774.0
R(int)[a]	0.0308
Data/restrains/parameters	5792/0/419
Completeness to θ (%)	99.2
Goodness-of-fit on F^2	1.061
R_1, wR_2 (I>2σ(I))[b]	0.0391/0.1052
R_1, wR_2 (all data)[b]	0.0441/0.1086
Largest diff. peak and hole (A^{-3})	1.50/-0.43
CCDC	2407090

[a] $R_{int} = \Sigma|F_o^2 - F_{o,mean}^2|/\Sigma F_o^2$, [b] $R_1 = \Sigma(||F_o| - |F_c||) / \Sigma|F_o|$; $wR_2 = [\Sigma w(F_o^2 - F_c^2)^2 / \Sigma w(F_o^2)^2]^{1/2}$

Table S2 Selected interatomic distances [Å] and angles [°] for the Cu(II) complex of **L2**-Boc

**Distances**		**Angles**	
Cu1–N1	1.980(2)	N1–Cu1–N2	84.48(8)
Cu1–N2	2.026(2)	N1–Cu1–N4	160.58(9)
Cu1–N4	1.995(2)	N2–Cu1–N4	82.94(8)
Cu1–O1	2.743(2)	N1–Cu1–O3	103.90(7)
Cu1–O3	1.997(1)	N2–Cu1–O3	158.84(8)
Cu1–O5	2.466(3)	N4–Cu1–O3	93.03(8)

Table S3

Fitted rate and equilibrium constants for transmetalation reaction of **MnL2** with Cu(II)

k_0 (s^{-1})	k_1 (M^{-1}s^{-1})	k_2 (M^{-2}s^{-1})	k_3 (M^{-1}s^{-1})	k_4 (M^{-2}s^{-1})	K_{MnLCu}
(0.1 ± 2.2)×10^{-3}	418 ± 300	(1.9 ± 1.0)×10^7	0.2 ± 0.1	–	83 ± 26

equation used for the data fitting:

$$k_{obs} = \frac{k_0 + k_1[\mathrm{H^+}] + k_2[\mathrm{H^+}]^2 + k_3[\mathrm{Zn^{2+}}] + k_4[\mathrm{H^+}][\mathrm{Zn^{2+}}]}{1 + K_{MnHL}[\mathrm{H^+}] + K_{MnLCu}[\mathrm{Zn^{2+}}]} \quad \text{(S1)}$$

Analysis of the ^{17}O NMR and ^{1}H NMRD data[1]

The reduced transverse ^{17}O relaxation rates, $1/T_{2r}$, and reduced ^{17}O chemical shifts, ω_r, are calculated from the measured relaxation rates $1/T_2$ and angular frequencies ω of the paramagnetic solutions and from the relaxation rates $1/T_{2A}$ and angular frequencies ω_A of the diamagnetic reference according to the Equations (S1) and (S2)[2]:

$$\frac{1}{T_{2r}} = \frac{1}{P_m}\left[\frac{1}{T_2} - \frac{1}{T_{2A}}\right] = \frac{1}{\tau_m}\frac{T_{2m}^{-2} + \tau_m^{-1}T_{2m}^{-1} + \Delta\omega_m^2}{(\tau_m^{-1} + T_{2m}^{-1})^2 + \Delta\omega_m^2} \quad \text{(S1)}$$

$$\Delta\omega_r = \frac{1}{P_m}(\omega - \omega_A) = \frac{\Delta\omega_m}{(1 + \tau_m T_{2m}^{-1})^2 + \tau_m^2\Delta\omega_m^2} \quad \text{(S2)}$$

$\Delta\omega_m$ is determined by the hyperfine or scalar coupling constant, $A_O/\hbar$, where B represents the magnetic field, S is the electron spin and g_L is the isotropic Landé g factor (Equation (S3)).

$$\Delta\omega_m = \frac{g_L\mu_B S(S+1)B}{3k_B T}\frac{A_O}{\hbar} \quad \text{(S3)}$$

The chemical shifts are measured with high errors (given the large linewidths) and the reduced chemical shifts were not further used in the fit.

The ^{17}O transverse relaxation rate is mainly determined by the scalar contribution, $1/T_{2sc}$, and it is given by Equation (S4).

$$\frac{1}{T_{2m}} \cong \frac{1}{T_{2sc}} = \frac{S(S+1)}{3}\left(\frac{A_O}{\hbar}\right)^2\left(\tau_{s1} + \frac{\tau_{s2}}{1+\tau_{s2}^2\omega_s^2}\right) \qquad \frac{1}{\tau_{si}} = \frac{1}{\tau_m} + \frac{1}{T_{ie}} \quad \text{(S4)}$$

The exchange rate, k_{ex}, (or inverse binding time, τ_m) of the inner sphere water molecule is assumed to obey the Eyring equation (Equation (S5)) where $\Delta S^\ddagger$ and $\Delta H^\ddagger$ are the entropy and enthalpy of activation for the exchange, and k_{ex}^{298} is the exchange rate at 298.15 K.

$$\frac{1}{\tau_m} = k_{ex} = \frac{k_B T}{h}\exp\left\{\frac{\Delta S^\ddagger}{R} - \frac{\Delta H^\ddagger}{RT}\right\} = \frac{k_{ex}^{298}T}{298.15}\exp\left\{\frac{\Delta H^\ddagger}{R}\left(\frac{1}{298.15} - \frac{1}{T}\right)\right\} \quad \text{(S5)}$$

The electronic relaxation is mainly governed by modulation of the transient zero-field splitting, and for the electron spin relaxation rates, $1/T_{1e}$ and $1/T_{2e}$, McMachlan has developed Equations (S6)–(S8):

$$\left(\frac{1}{T_{1e}}\right) = \frac{32}{25}\Delta^2\left(\frac{\tau_v}{1+\omega_S^2\tau_v^2} + \frac{4\tau_v}{1+4\omega_S^2\tau_v^2}\right) \quad \text{(S6)}$$

$$\left(\frac{1}{T_{2e}}\right) = \frac{32}{50}\Delta^2\left[3\tau_v + \frac{5\tau_v}{1+\omega_S^2\tau_v^2} + \frac{2\tau_v}{1+4\omega_S^2\tau_v^2}\right] \quad \text{(S7)}$$

$$\tau_v = \tau_v^{298} \exp\left\{\frac{E_v}{R}\left(\frac{1}{T} - \frac{1}{298.15}\right)\right\} \quad \text{(S8)}$$

where Δ^2 is the trace of the square of the transient zero-field-splitting (ZFS) tensor, τ_v is the correlation time for the modulation of the ZFS with the activation energy E_v, and ω_S is the Larmor frequency of the electron spin.

The proton relaxivities (normalized to 1 mM Mn^{2+} concentration) originate from inner- and outer-sphere contributions (Equation (S9)):

$$r_1 = r_{1is} + r_{1os} \quad \text{(S9)}$$

The inner-sphere term is given by Equation (S10), where q is the number of inner-sphere water molecules.

$$r_{1is} = \frac{1}{1000} \times \frac{q}{55.55} \times \frac{1}{T_{1m}^{H} + \tau_m} \quad \text{(S10)}$$

In the longitudinal relaxation rate of inner sphere water protons, $1/T_{1m}^{H}$, the dipolar contribution dominates (Equation (S11)):

$$\frac{1}{T_{1m}^{H}} \cong \frac{1}{T_1^{DD}} = \frac{2}{15}\left(\frac{\mu_0}{4\pi}\right)^2 \frac{\hbar^2 \gamma_S^2 \gamma_I^2}{r_{MnH}^6} S(S+1)\left[\frac{3\tau_{d1H}}{1+\omega_I^2 \tau_{d1H}^2} + \frac{7\tau_{d2H}}{1+\omega_S^2 \tau_{d2H}^2}\right] \quad \text{(S11)}$$

Here r_{MnH} is the effective distance between the Mn^{2+} electron spin and the water protons, ω_I is the proton resonance frequency, τ_{diH} is given by Eq. S12, where τ_{RH} is the rotational correlation time of the Mn^{2+}–H_{water} vector:

$$\frac{1}{\tau_{diH}} = \frac{1}{\tau_m} + \frac{1}{\tau_{RH}} + \frac{1}{T_{ie}} \qquad i = 1, 2; \quad \text{(S12)}$$

No scalar relaxation term was included in the analysis, as the NMRD profiles did not present a second, low-field dispersion, typical of an important scalar contribution to relaxivity.[1]

The outer-sphere contribution to the overall relaxivity is described by Equation (S13), where N_A is the Avogadro constant, and J_{os} is a spectral density function (Equation (S14)).

$$r_{1os} = \frac{32 N_A \pi}{405}\left(\frac{\mu_0}{4\pi}\right)^2 \frac{\hbar^2 \gamma_S^2 \gamma_I^2}{a_{MnH} D_{MnH}} S(S+1)\left[3J_{os}(\omega_I, T_{1e}) + 7J_{os}(\omega_S, T_{2e})\right] \quad \text{(S13)}$$

$$J_{\mathrm{os}}(\omega, T_{je}) = \mathrm{Re}\left[\frac{1 + \frac{1}{4}\left(i\omega\tau_{\mathrm{MnH}} + \frac{\tau_{\mathrm{MnH}}}{T_{je}}\right)^{1/2}}{1 + \left(i\omega\tau_{\mathrm{MnH}} + \frac{\tau_{\mathrm{MnH}}}{T_{je}}\right)^{1/2} + \frac{4}{9}\left(i\omega\tau_{\mathrm{MnH}} + \frac{\tau_{\mathrm{MnH}}}{T_{je}}\right) + \frac{1}{9}\left(i\omega\tau_{\mathrm{MnH}} + \frac{\tau_{\mathrm{MnH}}}{T_{je}}\right)^{3/2}}\right]$$

$$j = 1, 2 \qquad \text{(S14)}$$

The diffusion coefficient for the diffusion of a water proton away from a Mn^{2+} complex, D_{MnH}, obeys the exponential temperature dependence described by Equation (S15), with activation energy E_{MnH}:

$$D_{\mathrm{MnH}} = D_{\mathrm{MnH}}^{298} \exp\left\{\frac{E_{MnH}}{R}\left(\frac{1}{298.15} - \frac{1}{T}\right)\right\} \qquad \text{(S15)}$$

In the fitting procedure, some parameters have been fixed to common values: the distance between the metal ion and the inner and outer sphere water protons (r_{MnH} = 2.83 Å and a_{MnH} = 3.6 Å, respectively); the diffusion coefficient and its activation energy ($D_{\mathrm{MnH}} = 26\times10^{-10}$ m^2s^{-1} and E_{MnH} = 20 kJ mol^{-1}).

[1] E. Balogh, Z. He, W. Hsieh, S. Liu, É. Tóth; *Inorg. Chem.* 2007, **46**, 238-250.
[2] T. J. Swift, R. E. Connick, *J.Chem.Phys.* 1962, ***37,*** *2*, 307-320.
